# *Neisseria meningitidis*: a traditional extracellular pathogen with an intense intracellular lifestyle

**DOI:** 10.3389/fcimb.2025.1733264

**Published:** 2025-12-16

**Authors:** Silvia Caterina Resta, Adelfia Talà, Riccardo Conte, Matteo Calcagnile, Cecilia Bucci, Pietro Alifano

**Affiliations:** 1 Department of Experimental Medicine, University of Salento, Lecce, Italy; 2 Department of Biological and Environmental Sciences and Technologies, University of Salento, Lecce, Italy

**Keywords:** pathometabolism, meningococcus, host-pathogen interaction, intracellular lifestyle, nasopharyngeal barrier, blood-brain barrier

## Abstract

*Neisseria meningitidis* (meningococcus) is a transitory colonizer of the human nasopharynx that occasionally, for largely unknown reasons, reaches the bloodstream, translocating across the nasopharyngeal mucosa, causing septicemia. The bloodstream spread of bacteria to the meninges can cause meningitis after crossing the blood-brain barrier (BBB) and the blood-cerebrospinal fluid barrier (BCSFB). Thus, the meningococcus must cross several epithelial and endothelial barriers to cause invasive meningococcal disease (IMD). While meningococcal interactions on the surface of epithelial and endothelial cells have been intensively investigated, leading to the identification of key determinants of virulence of this bacterium, relatively little is known about the crossing of the nasopharyngeal epithelial barrier (NEB), the BBB, and BCSFB by the meningococcus. Several mechanisms (transcellular and paracellular) have been proposed, including transcellular crossing and paracellular crossing that might be favored by an epicellular lifestyle of this bacterium. Little is also known about the prevalent (vacuolar or cytoplasmic) localization of *N. meningitidis* in infected epithelial and endothelial cells and the mechanisms adopted by this microorganism to survive and multiply in the intracellular environment. The purpose of this article is to collect and review what is actually known about the intracellular lifestyle of these microorganisms. The picture that emerges is that although it is traditionally considered an extracellular pathogen (despite its original name, Diplococcus intracellularis meningitidis [Weichseilbaum, 1887]), *N. meningitidis* engages in complex interactions with host cells in the intracellular microenvironment, involving signal transduction, membrane trafficking, cytoskeleton, metabolic cross-talk, and programmed cell death.

## 1 Introduction

*Neisseria meningitidis* (the meningococcus) is a Gram-negative diplococcus that transiently colonizes the nasopharynx of healthy subjects and occasionally causes invasive meningococcal disease (IMD) (Hill et al., 2010). Despite the availability of modern and effective vaccines and the optimization of therapeutic protocols, IMD, with its most frequent manifestations, sepsis and meningitis, continues to claim victims and to represent a major public health problem. On a global scale, from 5 to 15% of the population is estimated to be composed of asymptomatic nasopharyngeal carriers of *N. meningitidis*, and less than 1% of colonized individuals develop IMD (World Health Organization, 2025). Incidence rates of meningococcal carriage and IMD are, however, strongly affected by age, population, geography, and time-related variations. The carriage rate is generally low in infants and children, while it increases and reaches its peak in adolescents. Differences in host genetics, demographic factors, social behaviors, circulating meningococcal strains, public health infrastructures, surveillance, and prophylactic interventions may explain differences in meningococcal epidemiology between different countries (Bogaert et al., 2005; Weckx et al., 2017; Kizil et al., 2021). For instance, in the USA, the carriage rate is around 24% (Peterson et al., 2019; Santos-Neto et al., 2019), in the African meningitis belt, it is between 10 and 20% but reaches 80% during outbreaks (Tefera et al., 2020), while in Asian countries it is generally low (1.5-9.1%) (Serra et al., 2020). However, despite the low carriage rate, in some Asian countries, the mortality rate of IMD is high due to weak surveillance and/or the lack of routine vaccination (Aye et al., 2020). IMD is characterized by high morbidity and mortality in children and adults. Lethality rates vary between 7 and 15% when the disease is treated but can exceed 50% when IMD is not treated (Wang et al., 2019). Rough estimations point to around 500,000 IMD cases worldwide, causing around 50,000 deaths each year (World Health Organization, 2025). Moreover, between 11 and 19% of patients who survive have severe sequelae, such as hearing loss, limb amputation, and neurological complications (Olbrich et al., 2018; Walter et al., 2021).

Key questions about meningococcal infection, which still await clear and exhaustive answers but are essential for identifying increasingly effective prophylactic and therapeutic strategies, are: i. How does the transition from asymptomatic colonization to disease occur? ii. Why is IMD so devastating? iii. What are the bacteria-, host-, and environment-related factors associated with increased incidence, severity, and mortality of meningococcal infection? iv. How did this bacterium, now considered an “accidental” pathogen, evolve? v. Is there a possibility of new pathogens emerging able to cause systemic disease in humans within the *Neisseria* genus? To address these questions, it is mandatory to understand the infection cycle of this bacterium.

To cause sepsis and meningitis, *N. meningitidis* has to cross cellular barriers. In fact, this common transitory colonizer of the human nasopharynx is able to cross the nasopharyngeal epithelial barrier (NEB), entering and replicating in the bloodstream, resulting in septicemia and/or septicemic shock. It is also able to cross the blood-brain barrier (BBB) to reach the subarachnoid space of the leptomeninges, causing meningitis with or without septicemia (van Deuren et al., 2000). The crossing of these cellular barriers is essential for the development of the IMD, and although it has been intensively studied, it remains not fully understood. Different mechanisms have been proposed in different cellular, organ, and animal models, including transcellular and paracellular routes. An intracellular stage of the meningococcal infection has also been identified, favored by specific bacterial factors differentiating it from other *Neisseria* spp. such as the polysaccharidic capsule (Nikulin et al., 2006; Spinosa et al., 2007), as well as other virulence determinants that will be reviewed here. Thus, *N. meningitidis* adds to the growing list of pathogenic bacteria traditionally classified as extracellular and now considered to have a dual intracellular/extracellular lifestyle (Silva, 2012; Casadevall and Fang, 2020). Indeed, cellular infection by meningococci and the complex interactions between bacteria and the host cell in the intracellular microenvironment are essential not only for the progression of the meningococcal infectious cycle but also for determining the signs and symptoms of the IMD. In this article, we will review what is actually known about these interactions, which involve signal transduction, membrane trafficking, the cytoskeleton, metabolic cross-talk, and programmed cell death.

## 2 *Neisseria meningitidis*: evolution and genetic features

Genomic and phylogenetic analysis indicate that all commensals and pathogenic *Neisseria* spp. evolved from a common rod-shaped ancestor (Chen et al., 2021; Nyongesa et al., 2022). This was adapted to mucosal colonization and likely unencapsulated (Clemence et al., 2018; Priniski and Seifert, 2018). The two human pathogens belonging to the genus *Neisseria*, *N. meningitidis* and *N. gonorrhoeae*, have evolved from a common more recent ancestor and share a high similarity with genetic identity of 80-90% (Bennett et al., 2014; Maiden and Harrison, 2016; Vigué and Eyre-Walker, 2019). However, the *N. meningitidis* population is much more diverse than the *N. gonorrhoeae* (Vigué and Eyre-Walker, 2019). Potential explanations are: i. lower effective population size of *N. gonorrhoeae* because it evolved from *N. meningitidis* and went through a bottleneck after speciation as a consequence of ecological isolation in the human genital tract (Vázquez et al., 1993) or because of the specificity of its biology; ii. lower mutation rates in *N. gonorrhoeae* population than *N. meningitidis* population, which is characterized by the frequent occurrence of mutator clones in disease-associated lineages (Bucci et al., 1999; Richardson and Stojiljkovic, 2001; Richardson et al., 2002; Colicchio et al., 2006a; Hall and Henderson-Begg, 2006; Omer et al., 2011); iii. less diversity acquired by DNA recombination in *N. gonorrhoeae* than in *N. meningitidis* (Vigué and Eyre-Walker, 2019).

Intriguingly, although *N. meningitidis* is a naturally transformable species, its population is structured into rather stable clonal complexes (ccs), which cluster genetically associated sequence type (ST) strains defined by multi-locus sequence typing (MLST) of seven conserved housekeeping genes (Maiden et al., 2013). Meningococci belonging to different ccs are associated differently with carriage or disease status. For example, meningococcal strains belonging to cc11 are rarely found as colonizers and are overrepresented in IMD, with an estimated disease/carriage ratio of 12.59, whereas cc53 generally behaves as a commensal with an estimated disease/carriage ratio of <0.1 (Mullally et al., 2021).

The ccs associated with IMD are termed hypervirulent lineages (Maiden, 2008). There is no core pathogenome to distinguish carriage from invasive *N. meningitidis* strains. In fact, even factors such as the capsule, which is a crucial virulence factor finely regulated, are associated with multiple ccs and can be present in carriage isolates (Oldfield et al., 2018; Whaley et al., 2022). However, it is worth noting that capsule null strains or strains with mutations impeding capsule expression are more frequently clustered in carriage-associated ccs, such as cc198, cc1136, and cc53 (Moreno et al., 2015; Neri et al., 2019; Olof et al., 2023).

Recently, other genes have emerged to be potentially enriched in invasive meningococcal isolates compared to carriage ones. Mullally and coworkers found genomic islands (GIs) associated with hyperinvasive lineages (absent in cc53), which encode functions that facilitate meningococcal access to different cell types, leading to an increased risk for IMD (Mullally et al., 2021). These GIs are involved in meningococcal adhesion and subsequent host cell invasion, iron uptake (*hpuAB*), meningococcal survival in the intracellular environment, modulation of the host cell cycle, innate immune escape and evasion of phagocytic killing, epigenetic control of gene expression (*modB*), and competition within the meningococcal population in the human nasopharynx. Among these GIs, Mullally and coworkers found the glutathione peroxidase-encoding gene *gpxA*, the capsular biosynthetic genes, and *pglI* coding for an enzyme involved in the O-acetylation of the pilin glycan (Mullally et al., 2021). This latter modification is known to affect the chain length of the pilin glycan on the surface of the pilus structure, which in turn modulates the interaction between pili and the immune system, but also affects the meningococcal adhesion and subsequent invasion of the host cell (Power et al., 2003; Mubaiwa et al., 2017).

Mullally and coworkers also noted that the autotransporter NadA has been acquired by a branch of the hyperinvasive common ancestor (Mullally et al., 2021). NadA is a surface protein and is involved in adhesion to epithelial cells (Capecchi et al., 2005), brain microvascular endothelial cells (Kulkarni et al., 2020) and binds with high affinity to sialic acid-binding immunoglobulin-type lectins (Siglec)-5 and Siglec-14, promoting bacterial invasion (Benucci et al., 2024). In another study, the gene encoding phage transposase NEIS1048 and the associated single-nucleotide polymorphisms (SNP) *glmU* S373C encoding the enzyme N-acetylglucosamine 1 phosphate (GlcNAc 1 P) uridyltransferase have been associated with invasive lineages. The latter is involved in the synthesis of UDP-N-acetylglucosamine pyrophosphorylase (UDP-GlcNAc), which is a substrate for the synthesis of lipooligosaccharide (LOS), capsule, and CMP-NANA, the substrate for sialic acid (Eriksson et al., 2023). Meningococcal carriage and invasive isolates have also displayed differences in subsequences, k-mers, of *fkbp*, *glmU*, *pilC*, and *pilE*, suggesting that variation in these genes could play a role in infection capability (Eriksson et al., 2023). PilE is, in fact, the core structural protein of Type IV pili, while PilC is the adhesin located at the top of the pilus, which is the most crucial factor for initial adhesion of meningococci to host cells (Virji et al., 1992; Forest and Tainer, 1997; Merz and So, 2000). In contrast, commensal colonizer lineages, such as cc53, have not acquired these GIs and are characterized by thirteen unique loss-of-function loci (Mullally et al., 2021).

Intriguing differences were also reported in the structure of a two-partner secretion system (TPS)-encoding locus, *tspA/tpsB* (also named *hrpA/hrpB*), between hyperinvasive ccs and cc53 (Mullally et al., 2021). TPS is a secretion pathway that appears to play diverse roles in several Gram-negative bacteria and includes a large secreted protein (generally referred to as TpsA) and a channel-forming β barrel outer membrane activator/transport protein (TpsB) (Henderson et al., 2004; Schielke et al., 2010; Jacob-Dubuisson et al., 2013; Willett et al., 2015). Meningococci contain up to three different TPS systems. System 1 (TpsA/TpsB, also known as HrpA/HrpB) appears to be meningococcal-specific, while systems 2 and 3 are overrepresented in disease isolates compared to carriage isolates, but are also present in *N. lactamica*, which also has a distinct system 4 that is absent in meningococci (van Ulsen et al., 2008). In *N. meningitidis*, the HrpA/HrpB TPS was implicated in diverse functions, including competition between meningococci as a toxin-antitoxin fratricide system, biofilm formation, adherence to epithelial cells, intracellular survival, vacuolar escape, interaction with dynein, and modulation of apoptosis/pyroptosis (Schmitter et al., 2007; Talà et al., 2008, Talà et al., 2022; van Ulsen et al., 2008; Neil and Apicella, 2009; Arenas et al., 2013).

The *tspA/tpsB* locus evolved in the hyperinvasive lineages with the appearance of repeated N-terminal-truncated *tpsA* genes (*tpsC* cassettes) and Immunity Open Reading Frames (IORFs), which are absent in cc53 (Mullally et al., 2021). It has been shown that low-frequency recombination with silent *tpsC* cassettes, which share sequence similarity with the central region of *tpsA* but show an entirely different 3’-terminal sequence, may introduce different toxic modules at the variable C-terminus of meningococcal TpsA (Arenas et al., 2013). Thus, the presence of the *tpsC* cassettes and the cognate IORFs could confer increased plasticity to a genomic region that appears to be involved in the evolution of virulence in meningococcus.

More importantly, as will be discussed below, because the genome content tends to be highly conserved between *bona fide* carriage and disease isolates, subtle differences in the ability to modulate gene expression in host microenvironments, to engage in productive cross-talk with the cells also at metabolic level, to elude the host immune defenses, and to evolve over a short period of time have been proposed as major determinants of the hyperinvasive phenotype. The extraordinary ability of *N. meningitidis* to evolve rapidly (microevolution) leads to considerable diversity among circulating meningococcal strains and a different ability of these strains to colonize the human host and cause IMD.

## 3 Meningococcal structures involved in the interactions with host cells, vacuole escape, and intracellular survival mechanisms

As a human-adapted pathogen, *N. meningitidis* has developed multiple structures to interact with the host cell and evade the immune system. These include structures and proteins specialized in entering the host cell and surviving in the intracellular environment, differentially expressed between strains more closely associated with the disease and strains associated with carrier status.

The first step in the infectious cycle of the meningococcus is the adhesion to the host cell. Initially, this is mediated by type IV pili, filamentous structures composed of the major pilin PilE and three minor pilins PilV, PilX, and ComP (Forest and Tainer, 1997; Maier et al., 2004). It has been shown that, following type IV pili-mediated contact, the *pilE* gene encoding the major pilin and the genes for capsule synthesis are downregulated, and this is thought to allow for more intimate adhesion through other outer membrane adhesins. The best studied of these adhesins are the opacity proteins Opa and Opc (Bhat et al., 1991; Hauck, 2003). The former is present in both the meningococcus and the gonococcus, while Opc is only present in the meningococcus. Additionally, a variety of other meningococcal outer membrane proteins fulfill the role of minor adhesins, such as NadA, NhhA, App, MspA, and HrpA of the meningococcal TPS system 1 HrpA/HrpB (Hadi et al., 2001; Scarselli et al., 2006; Schmitt et al., 2007; Nägele et al., 2011; Khairalla et al., 2015). Notably, all these minor adhesins appear to be meningococcal-specific and are not found in gonococci (Comanducci et al., 2002, Comanducci et al., 2004; Turner et al., 2006; Schielke et al., 2010).

These multiple interactions mediating the intimate adhesion of meningococci to the host cell led to the activation of host signaling pathways for the rearrangement of the host cytoskeleton to form protrusions that engulf the bacteria. The internalization frequency, however, is not fixed, but it depends on both the receptors expressed by the host cell and meningococcal factors. For instance, antigenic variability of the PorB porin affects its binding to TLR2 and consequently bacterial internalization (Toussi et al., 2016). At the same time, LOS sialylation, regulated by the availability of exogenous sialic acid, the regulation of genes for endogenous sialic acid production, and the expression of Lst sialyltransferase, enhance internalization in immune cells expressing Siglec-5 and sialoadhesin (Jones et al., 2003; Chang and Nizet, 2014) but inhibits Opc-mediated interaction with the host cell (de Vries et al., 1998).

Once internalized, it is mandatory for the meningococcus to avoid lysosomal killing; thus, it modifies the internalization vacuole via Lysosomal Associated Membrane Protein 1 (LAMP1) cleavage through IgA protease (Lin et al., 1997) and ultimately mediates the rupture of the vacuole. HrpA has been involved in this process because of its Mn^2+^-dependent hemolytic activity residing in its C-terminal domain (Talà et al., 2022). LAMP1 is one of the most abundant proteins of the lysosomal membrane. It is highly glycosylated and provides a protective barrier from the action of lysosomal hydrolases, thus maintaining lysosomal integrity (Eskelinen, 2006; Li and Pfeffer, 2016), in addition to playing a role in maintaining the lysosomal acidic pH (Zhang et al., 2023). Meningococci interaction through type IV pili has been shown to induce an increase in Ca^2+^ levels sufficient to redistribute LAMP1 to the plasma membrane, making it accessible to the IgA protease (Ayala et al., 2001). Meningococcal type IV pili interact with CD147 (Basigin/EMMPRIN) (Bernard et al., 2014; Maïssa et al., 2017) and with Platelet Activating Factor Receptor (PAFr) (Jen et al., 2013), as demonstrated in endothelial cells and upper airway epithelial cells, respectively. The interaction with these receptors is probably the reason for the increase in Ca^2+^ levels. In fact, platelet-activating factor binding to PAFr leads to the production of inositol trisphosphate (IP3) through the phosphatidylinositol cycle, which in turn binds to inositol 1,4,5-triphosphate receptor type 1 (IP3R1) to induce the release of Ca^2+^ stored in the endoplasmic reticulum (Kroegel et al., 1989; Mazer et al., 1991; Bito et al., 1992). CD147, on the other hand, activates the FAK-Scr pathway, leading to tyrosine phosphorylation of IP3R1, which enhances the affinity of the receptor for IP3 (Jayaraman et al., 1996; Tang et al., 2015).

**Figure 1:**
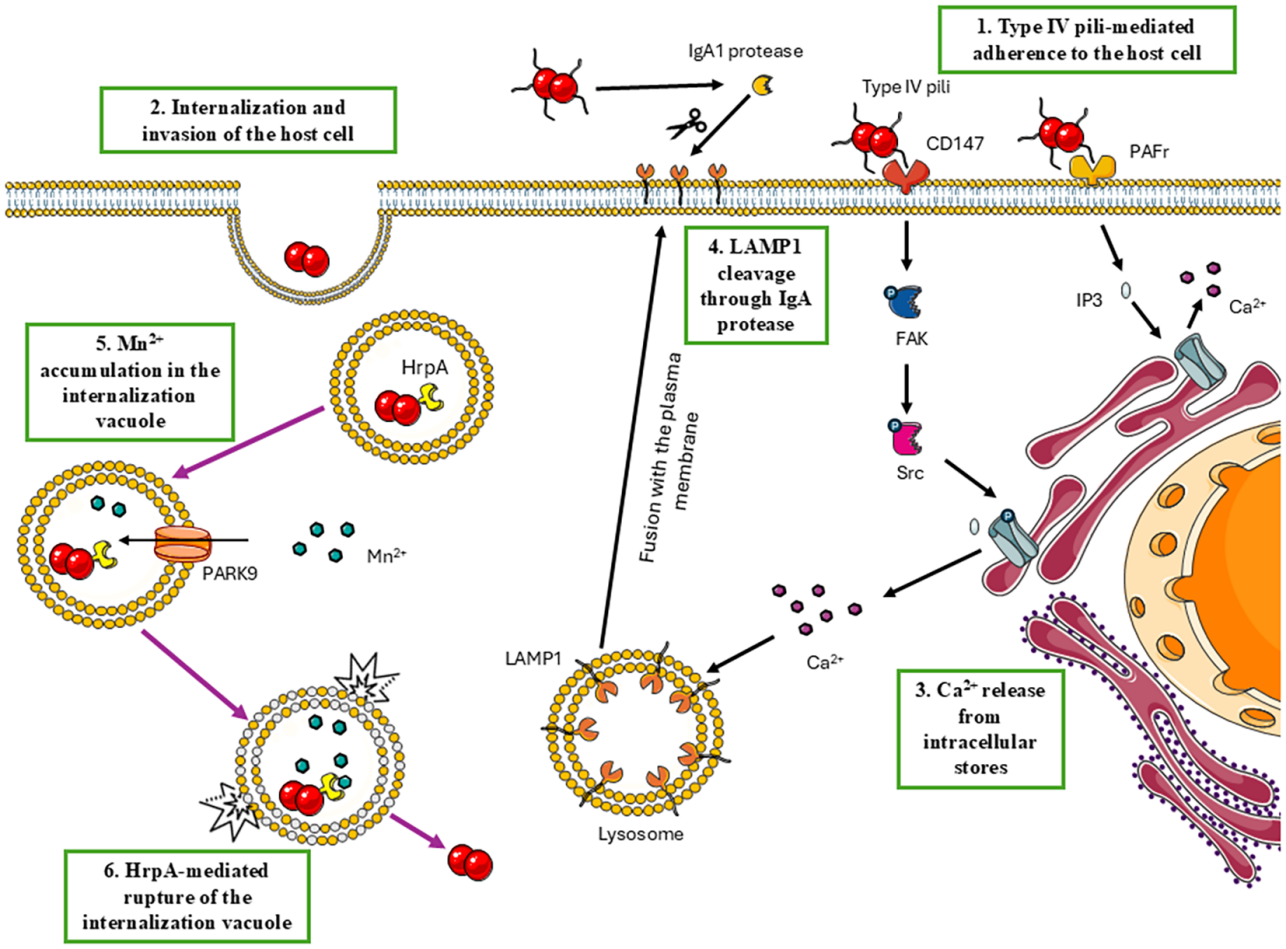
Proposed mechanism for *N. meningitidis* vacuolar escape. The meningococcus is internalized into the host cells in an endocytic compartment. Step 1 - Type IV pili-mediated interaction with CD147 and Platelet Activating Factor Receptor (PAFr). Step 2 – This interaction induces the formation of microvilli-like structures that engulf the bacteria and form an internalization vacuole. Step 3 – Type IV binding to CD147 and PAFr also induces the release of calcium from the storage of the endoplasmic reticulum via inositol trisphosphate (IP3) induction for PAFr signaling, and by increasing the affinity of IP3R1 for IP3 induced by CD147 signaling. Step 4 - Transient calcium increase induces the redistribution of LAMP1 to the plasma membrane, where it can be cleaved by meningococcal IgA protease, affecting the internalization vacuole maturation, and acidification. Step 5 - Meanwhile, PARK9 activity increases the vacuolar concentration of manganese ions. Step 6 – Mn^2+^ accumulation enables the hemolytic activity of the meningococcal TPS exoprotein HrpA, which ultimately breaks the vacuole. Adapted from Servier Medical Art (https://smart.servier.com), licensed under CC BY 4.0 (https://creativecommons.org/licenses/by/4.0/).

In the late endosomes and lysosomes, accumulation of Mn^2+^ may be obtained through the cation transporter ATP13A2 (PARK9), which has been identified as fundamental to prevent manganese toxicity, and proposed to shuttle manganese and other cations from the cytosol into the lysosomal lumen (Schmidt et al., 2009; Santoro et al., 2011; Nyarko-Danquah et al., 2020). Thus, meningococci-mediated depletion of LAMP1 potentially affects the internalization vacuole pH and its integrity. At the same time, the HrpA hemolytic activity is favored by the accumulation of Mn^2+^ in this compartment through PARK9. Overall, this can ultimately lead to the lysis of the vacuole, releasing meningococci into the cytosol. In agreement, meningococci deleted for IgA protease (Lin et al., 1997) or for HrpA (Talà et al., 2008) showed a dramatic reduction in survival/growth within different cell types. The proposed mechanism of meningococcal evasion from the internalization vacuole is illustrated in **Figure 1**.

Prevented from undergoing lysosomal degradation, the meningococcus must face the host defense mechanisms present in the intracellular environment. The capsule here plays a crucial role, probably protecting the bacterium from oxidative stress and antimicrobial peptides (Zaragoza et al., 2008; Brissac et al., 2021). Unencapsulated meningococci are, in fact, not able to survive inside the host cell, and capsule biosynthesis is upregulated in the intracellular environment (Spinosa et al., 2007). Spermidine has been shown to induce meningococcal capsule upregulation, increase meningococcal invasion by 5-fold, and enhance survival within macrophages (Kanojiya et al., 2022a). Spermidine is a polyamine whose concentration can reach the millimolar range in the intracellular environment, while extracellularly it is present in the low micromolar range (Heller et al., 1978; Lumkwana et al., 2022). Spermine, instead, is more easily found in the extracellular environment, and it has been shown to increase meningococcal adherence to epithelial cells and the expression of *pilE.* (Kanojiya et al., 2022b). Thus, meningococci may sense the intracellular environment through spermidine and increase capsule expression to survive in this microenvironment.

In the intracellular milieu, the meningococcus has been found to interact with Dynein Light Chain Tctex-Type 1 (DYNLT1) component of the motor protein dynein through a middle region of HrpA (Talà et al., 2022). This interaction enables the meningococcus to move along microtubules and come in contact with other organelles on this route, such as the mitochondrion (Talà et al., 2022). *N. meningitidis* can inhibit the intrinsic apoptosis pathway, translocating its porin PorB to the voltage-dependent anion channel (VDAC) on the mitochondrial outer membrane to prevent cytochrome c release (Massari et al., 2000, Massari et al., 2003). PorB-dependent apoptosis inhibition relies on HrpA-mediated meningococcal movement along microtubules, since infection in DYNLT1-silenced cells triggers apoptosis (Talà et al., 2022). Besides PorB, different meningococcal factors manipulate host cell death pathways. Among these, the autotransporters App and MspA can reach the nucleus, where they bind and cleave Histone H3 through their serine endopeptidase activity. This cleavage ultimately leads to a caspase-dependent cell death, which can be prevented by pan-caspase inhibitor Z-VAD-FMK (Khairalla et al., 2015). It has also been found that meningococcal IgA protease can translocate to the nucleus of the host cell because of the presence of a nuclear localization signal. Here, it cleaves the p65/RelA portion of NF-κB, inactivating it. Thus, the meningococcus can regulate NF-κB signaling, triggering its activation early in infection and then interrupting it. The signal, however, is not present in IgA protease of all meningococci but seems to be more represented in hyperinvasive strains (Besbes et al., 2015).

Meningococcal infections are characterized by a high inflammatory state (van Deuren et al., 1994; Møller et al., 2005; Zughaier, 2011; Ibrahim et al., 2024). This relies on the activation of signaling pathways upon the recognition of Pathogen-Associated Molecular Patterns (PAMPs) on bacteria or their Outer Membrane Vesicles (OMVs), mainly Toll-Like Receptors-4 (TLR4) and TLR2 signaling mediated by LOS (Christodoulides, 2021) and PorB recognition (Toussi et al., 2016), respectively. Moreover, PorB activation of TLR2 has been demonstrated to enhance bacterial internalization, suggesting that the expression of different PorB variants may be another factor affecting meningococcal internalization (Toussi et al., 2016).

OMVs are abundantly released from meningococci during infection and have been isolated in the blood and cerebrospinal fluid (CSF) of IMD patients (Stephens et al., 1982; Brandtzaeg et al., 1992). These vesicles are internalized through clathrin-mediated endocytosis, bringing multiple PAMPs into the host cell for the activation of inflammatory pathways. Key pathways activated include the canonical and non-canonical inflammasome. In the canonical pathway, NF-κB-dependent transcription of inflammasome components and their assembly is triggered by alterations in intracellular homeostasis. Consequently, pro-caspase-1 self-cleavage produces the activated caspase-1, which in turn cleaves gasdermin-D (GSDMD), and the N-terminal GSDMD fragments finally form pores into the plasma membrane through which inflammatory mediators are released, among which IL-1β and IL-18 (Franchi et al., 2009; Miao et al., 2010). In the non-canonical pathway, LOS on OMVs or intracellular bacteria is first recognized by guanylate-binding proteins (GBPs) that recruit caspase-4 (murine caspase-11) (Vanaja et al., 2016; Wandel et al., 2020). Activation of caspase-4 subsequently activates GSDMD, and the Damage-Associated Molecular Pattern (DAMPs) released can activate the inflammasome (Kayagaki et al., 2015; Liu et al., 2016). Excessive activation of these pathways leads to strong inflammation and death of the cell mediated by Ninjurin1 (NINJ1)-dependent cell lysis (Kayagaki et al., 2021), termed pyroptosis (Bergsbaken et al., 2009).

*N. meningitidis* activates both canonical and non-canonical pathways in the host, with predominance of the alternative caspase-3 mediated-GSDME activation *in vitro* in different cell lines (Talà et al., 2022) and of the GSDMD-dependent pathway *in vivo* in an intracisternal infected mouse model of meningitis (Pagliuca et al., 2024). Canonical pathway activation seems to be dependent on the intracellular localization of the bacterium. In fact, the infection with meningococci defective for HrpA/HrpB TPS is strongly impaired in the activation of caspase-1 and IL-1β and IL-18 release (Talà et al., 2022; Pagliuca et al., 2024), but if the integrity of the internalization vacuole is destabilized through DYNLT1-silencing (Driskell et al., 2007; Flores-Rodriguez et al., 2011; Yap and Winckler, 2022; Yap et al., 2022), caspase-1 activation occurs as in cells infected with wild-type meningococci (Talà et al., 2022).

Finally, the Cas9 protein of the Clustered Regularly Interspaced Short Palindromic Repeats (CRISPR)-Cas9 system has been implicated in meningococcal ability to adhere to and invade the host cell, and especially to survive in the intracellular environment (Sampson et al., 2013). The CRISPR-Cas9 system is normally used by bacteria as a defense mechanism against invading exogenous nucleic acid, but evidence suggests that this system has a more extensive function in bacterial physiology. For instance, in *Francisella novicida*, Cas9 has been found to repress expression of an endogenous mRNA (Sampson et al., 2013). Thus, meningococci may use the CRISPR-Cas9 system to regulate the expression of crucial factors for intracellular survival.

The final fate of intracellular meningococci is largely unknown, but it has been found that GPB1, mediating the initial assembly of GBP platform on cytosolic Gram-negative intracellular bacteria, and GBP3, a component of the GBPs recruited to the platform for caspase-4 activation, selectively lyse *F. novicida* and *N. meningitidis* (Feng et al., 2022). Therefore, the intracellular cytosolic cycle of meningococci may end with GBPs-mediated killing or with the release from the cell if pyroptosis occurs.

Overall, scientific efforts over the past twenty years have shed light on the behavior of meningococcus within the host cell and have supported a model in which, after internalization by the host cell, the meningococcus is able to avoid lysosomal killing by cleaving LAMP1 through IgA protease, and to escape from the internalization vacuole through the C-terminal hemolytic domain of HrpA, reaching the cell cytosol. Here, the meningococcus is able to sense the cytosolic environment, modulating the expression of the capsule and other virulence determinants. It exploits the microtubule cytoskeleton and dynein to move within the cell and interact with cellular structures, including mitochondria and the inflammasome. Using these mechanisms, which involve capsule, LOS, type IV pili, IgA protease, HrpA, PorB and other virulence determinants, the meningococcus is able to survive/multiply within infected cells and modulates the balance between apoptosis and pyroptosis.

## 4 From meningococcal colonization to invasion: nasopharyngeal epithelial barrier crossing

### 4.1 Interaction between *N. meningitidis* and epithelial cells

*N. meningitidis* resides as a colonizer in the human nasopharynx, which is its unique environmental niche (Coureuil et al., 2019). This site is characterized by a pseudostratified columnar ciliated epithelium composed of ciliated, basal, secretory, and goblet cells, which are responsible for mucus production, all held together by tight and adherens junctions. The nasopharynx is also characterized by macrophages, lymphocytes, and dendritic cells, embedded in the epithelial and submucosal layers. The abundance and differentiation state of the cells and the barrier function of the nasopharynx change with age and in response to different environmental issues, making it a dynamic barrier that combines physical, cellular, and molecular defenses to preserve respiratory health (Hill et al., 2010; Coureuil et al., 2019; Zhou et al., 2020; Hernandez-Leyva et al., 2025). Interaction of epithelial cells with meningococcal type IV pili and other minor adhesins triggers the polymerization of cortical actin and the clustering of ICAM1, CD44, EGFR, and ezrin underneath the microcolony, ultimately forming a cortical plaque (Merz et al., 1999). The latter is a structure formed by the local rearrangement of host cytoskeleton enriched in the aforementioned proteins that gives the bacterium an anchoring point and enables the triggering of the signaling required for meningococcal internalization (Merz et al., 1999). Ezrin belongs to Erzin, Radixin, Moesin (ERM) proteins. These, in their active form, connect the actin cytoskeleton with the plasma membrane, orchestrating the rearrangements of the membrane, such as the formation of microvilli. The N-terminal of active ERM proteins interacts with the cytosolic domain of the transmembrane CD44 and ICAM-1 proteins, while the C-terminal domain is bound to F-actin (Bretscher, 1999; Ramalho et al., 2020). Finally, the cortical plaque develops in microvilli-like protrusions that engulf the bacterium.

The Calu3 cell line is the most widely used model to reproduce the upper respiratory epithelium, as it is able to recapitulate differences in mucus layer thickness and cytokine production when grown under liquid-liquid interface (LLI) or air-liquid interface (ALI) conditions. The 8013 hyperinvasive meningococcus strain remains trapped and survives in the mucus layer in Calu3 grown on ALI (Audry et al., 2019), although in another study, the same strain was also able to transverse the Calu3 ALI model without altering the Trans-Epithelial Electrical Resistance (TEER) or the localization of tight junction proteins ZO-1 and occludin (Peters et al., 2024), suggesting the use of a transcellular route. The authors further use the invasive isolates MC58 and DE13664, and the carrier isolates α711 and α275, revealing a dramatic change in the capability of crossing the Calu3 monolayer and in the number of intracellular bacteria between invasive and carrier isolates (Peters et al., 2024). On the other hand, Dave and coworkers found a disruption of the Calu3 ALI monolayer infected with R001, N222.1, and N459.6 meningococcal strains, suggestive of a paracellular route of epithelial crossing (Dave et al., 2023). In particular, the carrier strains N222.1, R001, B285, R191, N459.6, and N59.1 were found to be able to increase the permeabilization of the Calu3 ALI barrier with disruption of the tight junction as demonstrated by the discontinuous occludin staining. However, the same authors found that the carrier N59.1 and N459.3 strains and the invasive strain MC58 did not affect the barrier function of the Calu3 ALI monolayer (Dave et al., 2023). Not surprisingly, the absence of a thick mucus layer in the Calu3 LLI model led to a higher transmigration rate for MC58 and 8013 strains compared to the ALI culture (Peters et al., 2024).

When sought, intracellular meningococci were found inside the cells, despite the transcellular or paracellular route followed, which opens the possibility that the intracellular phase of meningococcal infection may also contribute to the paracellular route. In agreement, the deletion of *pilE* strongly impairs both the survival/growth of meningococci in the intracellular environment and the capability of the bacterium to alter the barrier function of the Calu3 ALI model (Dave et al., 2023). In the Calu3 LLI model, it has also been clearly demonstrated that the MC58 strain uses the transcellular route of infection (Sutherland et al., 2010). Deletion of *pilE* was again found critical for intracellular growth/survival and for the transcellular crossing. Moreover, in agreement with the critical role of the capsule for meningococcal intracellular survival (Spinosa et al., 2007), *siaD* deletion in the MC58 strain led to an increase in adhesion to Calu3 LLI but to a strong reduction in intracellular bacteria and in transcellular crossing (Sutherland et al., 2010). Recently, primary cells from the mucoid tissue of the nose septum, the nasal epithelium of the nose cavity, or the concha inferior grown on the ALI system were tested for barrier function after meningococcal infection (Arends et al., 2025). The authors used different meningococcal strains belonging to cc11 and cc22, which were found to be able to decrease the TEER of the epithelium formed by primary cells from the mucoid tissue of the nose septum or the nasal epithelium of the nose cavity, but not of that formed by primary cells from the concha inferior (Arends et al., 2025). Moreover, meningococci belonging to cc11 were found to have a slightly greater impact on epithelial permeability (Arends et al., 2025). Thus, as summarized in **Table 1**, the potential crossing route exploited seems to be dependent on the meningococcal strain, with strains belonging to cc32 and cc22 more likely to follow the transcellular route, while those belonging to cc11 more likely to follow the paracellular route.

**Table 1:** Proposed traversal pathways exploited to cross different upper respiratory tract epithelium models by different meningococcal strains.

Strain	MLST designation	Nasopharyngeal epithelial model	Crossing route suggested	Intracellular meningococci	Reference
8013	C: P1.21,26-2: F1-5: ST-177 (cc18)	Calu3 (ALI)	- No crossing - Transcellular	- Not addressed - Not addressed	(Audry et al., 2019) (Peters et al., 2024)
MC58	B: P1.7,16-2: F1-5: ST-74 (cc32)	Calu3 (ALI) Calu3 (LLI)	- Transcellular - Transcellular - Transcellular	- Yes - Not addressed - Yes	(Peters et al., 2024) (Dave et al., 2023) (Sutherland et al., 2010)
DE13664	W: P1.18-1,3: F4-1: ST-22 (cc22)	Calu3 (ALI)	- Transcellular	- Yes	(Peters et al., 2024)
α711	B: P1.7,16: F3-3: ST-32 (cc32)	Calu3 (ALI)	- Transcellular	- Yes	(Peters et al., 2024)
α275	W: P1.18-1,3: F4-1: ST-22 (cc22)	Calu3 (ALI)	- Transcellular	- Yes	(Peters et al., 2024)
R001	W: P1.5,2: F1-1: ST-11 (cc11)	Calu3 (ALI)	- Paracellular	- Yes	(Dave et al., 2023)
B285	W: P1.5,2: F1-1: ST-11 (cc11)	Calu3 (ALI)	- Paracellular	- Yes	(Dave et al., 2023)
R191	W: P1.5,2: F1-1: ST-11 (cc11)	Calu3 (ALI)	- Paracellular	- Yes	(Dave et al., 2023)
N222.1	Y: P1.5-1,10-1: F4-1: ST-1655 (cc23)	Calu3 (ALI)	- Paracellular	- Not addressed	(Dave et al., 2023)
N459.3	Y: P1.5-1,10-1: F4-1: ST-1655 (cc23)	Calu3 (ALI)	- Transcellular	- Not addressed	(Dave et al., 2023)
N459.6	Y: P1.5-1,10-1: F4-1: ST-1655 (cc23)	Calu3 (ALI)	- Paracellular	- Not addressed	(Dave et al., 2023)
N59.1	Y: P1.21,16: F3-7: ST-1466 (cc174)	Calu3 (ALI)	- Transcellular	- Not addressed	(Dave et al., 2023)
2121218 2181243 2161142 2150374 2170011	W: P1.5,2: F1-1: ST-11 (cc11) W: P1.18-1,3: F4-1: ST-3422 (cc22)	Primary cells from the mucoid tissue nose septum or from the nasal epithelium of the nose cavity (ALI)	- Paracellular	- Not addressed	(Arends et al., 2025)

The transcellular crossing of the epithelium exploits different components of the host intracellular trafficking system. Vesicles positive for Rab11, Rab22a, and Rab3, orchestrating different steps in vesicle trafficking, are mislocalized to the cell periphery and enclose meningococci (Barrile et al., 2015). In addition, cytoskeleton dynamics are critical. Disruption of microtubules or inhibition of their dynamical depolymerization reduced transcellular crossing of meningococci without affecting intracellular replication (Sutherland et al., 2010; Barrile et al., 2015). The disruption of the Trans-Golgi Network (TGN) also leads to a reduced transcellular crossing, while the disruption of the actin cytoskeleton completely abolishes transcellular crossing (Barrile et al., 2015), probably because actin plays a crucial role in the engulfment of the bacteria.

Regardless of the method used to cross the epithelium, *N. meningitidis* encounters and must resist the underlying phagocytic cells, which are primarily represented by dendritic cells and resident macrophages, as well as the recruited neutrophils. Macrophages and neutrophils recognize and phagocytose opsonized bacteria (van Lookeren Campagne et al., 2007; Krüger et al., 2018), but the presence of the capsule (Jarvis and Vedros, 1987; Agarwal et al., 2014), the factor H binding protein (fHbp) (Lewis et al., 2012), the sialylation of LOS (Estabrook et al., 1997), and the IgG3 degradation through IgA protease type 1 (Spoerry et al., 2021) protects the meningococcus from complement deposition. In particular, IgG3 degradation inhibits their clustering and the recruitment of C1, and, therefore, the activation of the classical pathway (Spoerry et al., 2021). The capsule polysaccharide from serogroups A, B, C, W, and Y meningococci inhibits C1q interaction with bound anti-fHbp and anti-porin A antibodies, and, consequently, the deposition of C4b and classical complement pathway (Agarwal et al., 2014). Moreover, polysialic capsule of serogroups B and C meningococci (Jarvis and Vedros, 1987), as well as LOS sialylation (Vogel et al., 1997), limits C3 deposition, inhibiting the alternative pathway. A further interference with the C3 deposition is the binding of the regulator of the alternative pathway factor H by fHbp (Lewis et al., 2012). Nevertheless, macrophages and dendritic cells can phagocytize non-opsonized meningococci, so the bacterium developed strategies to evade intracellular killing and interfere with the immune function of these cells. For instance, the nitric oxide reductase NorB and the cytochrome c’ (CycP) detoxified NO produced by macrophages (Stevanin et al., 2005), while PorB-TLR2 interaction enhances anti-inflammatory IL-10 production (Samarasinghe et al., 2006) and modulates Nf-κB signaling (Oliveira-Nascimento et al., 2012), reprogramming dendritic cells toward a Th2 response (Mukherjee et al., 2016). Dendritic cells and macrophages secreted TNF-α early after meningococcal infection (Kolb-Maourer et al., 2001; Pridmore et al., 2001), and IL-1β, which is also produced by neutrophils (Kolb-Maourer et al., 2001; Idosa et al., 2019), among many other pro-inflammatory cytokines. The inflammatory environment is known to alter junctional proteins and barrier function of the epithelium. In fact, TNF-α, especially when associated with IFN-γ, dramatically reduced ZO-1 and JAM expression and localization in the airway epithelium (Coyne et al., 2002). IL-1β, on the other hand, has been shown to interfere with the ion selectivity of tight junctions (Coyne et al., 2002), and, in combination with High Mobility Group Box 1 (HMGB1), induces the downregulation of occludin and claudin-1 and the mislocalization of E-cadherin and β-catenin (HUANG et al., 2016).

Therefore, a combination of transcellular and paracellular routes for crossing the nasopharyngeal barrier is also possible if the meningococci crossing via the transcellular route induce a sufficient level of inflammation to alter epithelial permeability, allowing other bacteria to follow the paracellular route.

One alternative possibility suggested for *N. meningitidis* is the direct invasion of the central nervous system (CNS) through the olfactory nerve, bypassing the nasopharyngeal barrier. The first evidence of the exploitation of this route was obtained by Sjölinder and Jonsson in human CD46 transgenic mice (Sjölinder and Jonsson, 2010). Of the intranasally challenged mice, 20% developed meningitis without bacteremia, and the authors found that the infection induced a decreased expression and a relocation of the junction protein N-cadherin in the olfactory epithelium, which connects the nasopharynx with the CNS. Meningococci were found associated with the olfactory epithelium, in the submucosa, along the olfactory nerves, and the meninges (Sjölinder and Jonsson, 2010). The movement of the meningococcus along microtubules mediated by HrpA-DYNLT1 interaction may be relevant for this route of infection. In fact, *in vitro*, HrpA-deficient meningococci were impaired in reaching the neurites of the cells, accumulating in the cell body (Talà et al., 2022). The olfactory epithelium, besides olfactory nerves, houses the trigeminal nerve terminations. The trigeminal nerve branch is another direct route from the nose to the CNS, exploited by some pathogens, such as *Streptococcus agalactiae*, responsible for neonatal meningitis, and *Burkholderia pseudomallei*, responsible for meliodosis (St. John et al., 2016; Chacko et al., 2022).

*N. meningitidis* has been shown to be phagocytized by primary mouse trigeminal Schwann cells without causing the death of these cells (Delbaz et al., 2020). Instead, it induces alterations in protein expression patterns associated with cell-cell interactions and cellular movement (Delbaz et al., 2020), suggesting that the trigeminal nerve may also serve as an alternative pathway for the bacterium to reach the CNS from the nasal cavity.

### 4.2 Interaction between *N. meningitidis* and the nasopharyngeal microbiota

The human nasopharynx also hosts a complex, dynamic microbial community that is subject to variations related to host genetics, age, environment, life-style, and geographic location. This community plays a critical role not only in defense against pathogen and in immune system modulation, but also in the course and outcome of infections by modulating the host-pathogen response (Biesbroek et al., 2014; Esposito and Principi, 2018; Flynn and Dooley, 2021). The microbial community is dominated by six main genera: *Haemophilus*, *Streptococcus*, *Moraxella*, *Alloiococcus* and *Corynebacterium* (Bogaert et al., 2011; Stearns et al., 2015; Teo et al., 2015). The impact of the nasopharynx microbiota on the growth, survival and expression of virulence determinants of *N. meningitidis* is, however, largely unexplored, especially by experimental approaches, as highlighted in a recent systematic review (Yu et al., 2025). An initial study investigating the nasopharyngeal microflora of the affected population during outbreaks of serogroup A meningococcal disease in Seattle, Washington, and Portland, Oregon, found that the presence of *Staphylococcus epidermidis*, *Streptococcus MG-intermedius*, *Streptococcus morbillorum* (now renamed *Gemella morbillorum*), *Streptococcus sanguinis*, *Streptococcus mitis*, and several *Lactobacillus* and *Bacillus* species may be associated with resistance to acquisition of meningococci or to meningococcal disease (Filice et al., 1985).

Regarding *Lactobacillus* spp., several vaginal species such as *Lactobacillus crispatus* and *Lactobacillus gasseri* are common in the nasopharynx of naturally born infants and are still present in adults although to a lesser extent (Bogaert et al., 2011; Dzidic et al., 2018). It has recently been shown that *Lactobacillus crispatus* enhances *N. meningitidis* lysosomal killing *in vitro* and reduces meningococcal epithelial transmigration rate (Lidberg et al., 2025b). Moreover, *L. crispatus* has been shown to coaggregate with *N. meningitidis* microcolonies through interaction with meningococcal pili, interfering with microcolonies stability and meningococcal infection dynamics (Lidberg et al., 2025a). These findings may explain the protective effect of *Lactobacillus* spp. colonization on meningococcal colonization and disease (Filice et al., 1985).

Instead, the negative correlation between group A meningococcal acquisition or disease and nasopharyngeal colonization by *Streptococcaceae* may be imputed to the ability of streptococcal pyruvate oxidase (SpxB) to produce large amount of hydrogen peroxide that inhibits the growth of *N. meningitidis* (Pericone et al., 2000; Okahashi et al., 2014). However, a more recent study demonstrates that *S. mitis* coinfection enhanced the growth of a serogroup C strain of *N. meningitidis* (NME 8013) in a model with the Calu3 cell line, used to model the human airway epithelium, grown in air interface culture (cells grown with the apical domain facing air) to stimulate mucus production (Audry et al., 2019). This property has been partially attributed to the ability of *S. mitis* to hydrolyze glycan in the mucus, releasing sialic acid, a potential additional nutrient source, although meningococci were unable to grow in the presence of sialic acid as the sole carbon source, and the addition of sialic acid to the mucus of Calu3 cells was not sufficient to enhance meningococcal growth (Audry et al., 2019).

In particular, the study by Audry and colleagues (Audry et al., 2019), using the air interface cell culture model with a polarized mucus-secreting epithelium, proposes that *N. meningitidis* does not invade epithelial cells, but, rather, remains trapped within the mucus layer being unable to secrete enzymes capable of degrading the mucus, which protects meningococci from dehydration. Thus, it has been proposed that the nasopharyngeal mucus layer is the natural niche of meningococci, which may reside in this microenvironment as colonizers without inducing inflammation. Meningococci may be able to invade epithelial cells in areas where the mucus layer is interrupted possibly due to inflammation or the activity of the resident microbiota that is able to attack the mucus, for instance by hydrolysis of glycans (Audry et al., 2019). Notably, many commensal Streptococcus species of the colonizing the human oral cavity and the pharynx, including *Streptococcus anginosus*, *S. mitis*, *Streptococcus mutans*, *Streptococcus oralis*, *S. sanguis*, and *Streptococcus sobrinus* have mucin-degrading enzymes, and, in particular, *S. mitis* and *S. oralis* have neuraminidase activity (Derrien et al., 2010).

Neuraminidase activity is present also in *Streptococcus pneumoniae*, and it has been proposed that this activity increases the adhesion to epithelial cells of meningococci containing sialic acid in their LOS and/or capsules (serogroup B, C, Y and W135) by degrading the surface-exposed sialic acid and unmasking the meningococcal adhesins (Shakhnovich et al., 2002). A similar mechanism has been proposed for the neuraminidase from the influenza A virus (Rameix-Welti et al., 2009). On the other hand, a moderate inhibitory effect of *S. pneumoniae* on the growth of *N. meningitidis* was also observed *in vitro*, due to hydrogen peroxide production by *S. pneumoniae* (Pericone et al., 2000). Additionally, it is worth noting that streptococci are lactic acid bacteria and that lactate is a major carbon source for meningococcal growth (see below). Overall, these results demonstrate that the interactions between streptococci and meningococci are complex, dependent on numerous factors, and that the outcome of such interactions may be strictly dependent on the streptococcal species or strain, the meningococcal serogroup or strain, and their metabolic capabilities.

Regarding the other pathogens, an observational study assessing nasopharyngeal carriage of five bacteria (*S. pneumoniae*, *Haemophilus influenzae*, *Moraxella catarrhalis*, *Staphylocuccus aureus*, and *N. meningitidis*) in febrile children with and without acute respiratory infection (ARI) of the upper (URTI) or lower tract demonstrated that carriage of *M. catarrhalis* did not affect carriage of *S. aureus* or *N. meningitidis*, in contrast to both *S. pneumoniae*, *H. influenzae* which were both positively associated with *N. meningitidis* and negatively associated with *S. aureus* carriage (Chochua et al., 2016). However, there is currently no experimental study available investigating the interactions between *N. meningitidis*, *H. influenzae*, *S. aureus* and *M. catarrhalis* using co-culture or co-infection experiments (Yu et al., 2025).

Other members of the nasopharyngeal microbiota affect *N. meningitidis* survival and colonization in this environment, including other *Neisseria* spp. *Neisseria cinerea* has been shown to compete with the meningococcus for adhesion to epithelial cells, impairing meningococcal microcolonies formation (Custodio et al., 2020) while *N. lactamica* colonization is associated with reduced meningococcal carriage (Gbesemete et al., 2019). As discussed below, *N. lactamica* carriage is highly prevalent in young children peaking around age two and decreases with age (Gold et al., 1978; Cartwright et al., 1987; Bennett et al., 2005), concomitant with an increase in the abundance of bacteria producing propionic acid, an organic acid toxic to *N. lactamica* but not to *N. meningitidis* that is able to utilize propionic acid as an additional carbon source (Catenazzi et al., 2014). In fact, propionic acid-producing bacteria, such as *Fusobacterium nucleatum*, have been positively correlated with meningococcal abundance (Retchless et al., 2020), in line with the proposed propionic acid cross-feeding mechanism (Catenazzi et al., 2014). Regarding this cross-feeding mechanism, it is worth noticing a study showing that *Propionibacterium acnes* (now renamed *Cutibacterium acnes*) was among the top 10 taxa identified by 16 S rRNA pyrosequencing in chronic rhinosinusitis (CRS) patients (Hauser et al., 2015), while another study that characterized the nasopharyngeal microbiota in healthy subjects and during rhinovirus challenge correlated the presence of *Neisseria* with those of *Propionibacterium* (Allen et al., 2014). This correlation may assume particular significance because *P. acnes* produces propionic acid during anaerobic metabolism and some subspecies encodes a surface neuraminidase (McDowell et al., 2016; Yu et al., 2022), and may be thus involved in metabolic interactions with meningococci at the level of the nasopharynx. This hypothesis is speculative, but challenging. Future work will shed light on these aspects.

Finally, no competition but, on the contrary, cooperation was observed between *N. meningitidis* strains *in vitro* using the double-strain biofilm system, despite the fact that they produce the fratricide toxins TpsA and MafB (Pérez-Ortega et al., 2017), although it can be noted that colonization of the nasopharynx by different meningococcal strains is very rare and detected only in approximately 1% of carriers (Caugant et al., 2007). TpsA1 toxin acts by contact-dependent inhibition (CDI) (Poole et al., 2011; Arenas et al., 2013; Jamet et al., 2015), and, as previously indicated, it is thought to be involved in competition between meningococcal strains that possess different toxins and IORFs for niche colonization. Despite the numerous interactions between *N. meningitidis* and nasopharyngeal microbiota, IORFs do not appear to be influenced or influence other bacterial species. However, the work of Pérez-Ortega and co-workers (Pérez-Ortega et al., 2017), although it uses only two meningococcal strains, highlights the need to investigate the conditions in which this fratricide system can take place.

In summary, although the process of meningococcal interaction with epithelial cells has been studied for many years and many meningococcal adhesins/invasins have now been characterized in detail, much remains to be clarified about the relevance, especially *in vivo*, of the results obtained with *in vitro* cellular models. Critical points are represented by the actual relevance of the mucus layer covering the epithelium of the nasopharynx, by the interaction of the meningococcus with the different cell types of the human nasopharynx and the local microbiota, and by the mechanism of invasion of the epithelium (intracellular or paracellular). The interaction of the bacterium with the mucus layer is the subject of more recent studies using cell culture models that closely mimic the conditions of the nasopharynx *in vivo*. These models have recently provided new information on the colonization of the mucus layer by meningococcus and its interaction with the nasopharyngeal microbiota and other pathogens, which appears to play an important role in meningococcal colonization and the development of IMD, although further investigation is needed. Regarding the mechanisms of crossing the nasopharyngeal barrier, a combination of transcellular and paracellular pathways seems possible if meningococci using the transcellular pathway induce a sufficient level of inflammation to alter epithelial permeability, allowing other meningococci to follow the paracellular pathway. The ability to follow transcellular pathways and induce inflammation can vary greatly among meningococcal strains and, therefore, the discrepancies found in the literature between transcellular and paracellular pathways for mechanisms for crossing the nasopharyngeal barrier may be due to the use of different strains in the various studies, as well as different cellular models. Finally, the possibility arises that meningococcus may use the olfactory and trigeminal nerves to reach the CNS directly from the nose, bypassing the bloodstream, but this intriguing hypothesis requires further support.

## 5 How to reach the central nervous system: blood-brain barrier and blood-cerebrospinal fluid barrier traverse

The brain is normally protected from exogenous insults, such as pathogenic bacteria and toxic substances, through the meninges, the blood-brain barrier (BBB), and the blood-cerebrospinal fluid barrier (BCSFB). The meninges envelop the Central Nervous System (CNS) and are composed of three membranes: from the outermost, the dura mater, the arachnoid mater, and the pia mater (Kadry et al., 2020). Between the arachnoid membrane and the pia mater, there is the subarachnoid space, which contains cerebrospinal fluid (CSF) produced by the choroid plexuses (Menéndez González, 2023). The BBB regulates the passage of ions, nutrients, and oxygen from the blood to the brain and consists of endothelial cells supported by astrocytes and pericytes. The barrier function is established by the lack of fenestrae in the vessels, the tight junctions connecting the endothelial cells, which prevent the paracellular route for external agents, and the low level of transcytotic vesicles (Rodrigues and Granger, 2015). The stability of tight junctions between endothelial cells is guaranteed by interactions with pericytes (Jo et al., 2013), and by Wnt proteins, vascular Endothelial Growth Factor (VEGF), and Transforming Growth Factor-β (TGF-β), released by astrocytes (Guérit et al., 2021; Pivoriūnas and Verkhratsky, 2021; Sobral et al., 2025). Pericytes are also crucial for the suppression of transcytosis. This is achieved through the induction of endothelial expression of Major Facilitator Superfamily Domain-containing 2a (Mfsd2a), which is a transporter of docosahexaenoic acid (DHA) (Ben-Zvi et al., 2014; Nguyen et al., 2014). The incorporation of DHA creates a unique lipid composition of brain endothelial membranes, suppressing the formation of caveolae (Nguyen et al., 2014). A perivascular space, located between endothelial cells and astrocytic endfeet, can be present. This, often absent in capillaries, is called the Virchow-Robin perivascular space, and it is filled with interstitial fluid that has lymphatic functions (Esiri and Gay, 1990).

The BCSFB barrier is composed of an ependymal epithelium that produces the CSF, characterized by apical tight junctions located at the choroid plexuses. Choroid plexuses are ventricular structures that surround a stroma rich in fenestrated capillaries (Damkier and Praetorius, 2020). Postmortem examination of patients with *purpura fulminans* and IMD revealed meningococcal microcolonies associated with the endothelium of different vessels of the two barriers, such as the capillaries of the choroid plexuses, meningeal vessels, and brain parenchyma vessels (Mairey et al., 2006; Dando et al., 2014; Ridpath et al., 2014). This supports the hypothesis that meningococcal translocation across brain barriers occurs primarily through the endothelium, without the active participation of immune cells. Notably, no bacteria were observed in contact with the ependymal epithelium of the choroid plexuses, suggesting that meningococcus does not cross this barrier (Dando et al., 2014).

A deeper understanding of the dynamics of meningococcal colonization of different vascular beds was possible thanks to the use of a mouse model grafted with human skin (Manriquez et al., 2021). Similar to what was observed in human patients, the meningococcus colonized capillaries, venules, and arterioles with the progressive accumulation of recruited neutrophils in the humanized mouse model. Neutrophil recruitment was found to be subordinate to the Type IV pili-dependent adhesion to the endothelium, and it was crucial in controlling the number of adherent meningococci (Manriquez et al., 2021). In fact, neutrophils were able to detach meningococci bound to the endothelium and phagocytize them. However, *N. meningitidis* rapidly colonized capillaries and arterioles before the activation of an efficient inflammatory response. Neutrophils fail to translocate into capillaries and arterioles because of the absence of adhesion molecules such as E-selectin on these vessels (Manriquez et al., 2021). Thus, these can be used by the meningococcus to evade detection by neutrophils, allowing the infection to proceed.

Meningococcal adhesion and invasion of endothelial cells have proven to be a prerequisite for further dissemination. It has been demonstrated that at least 10 bacteria per microcolony on endothelial cells are needed to induce the recruitment of ezrin and the consequent cytoskeletal remodeling leading to the internalization of the bacteria (Soyer et al., 2014). Meningococcal internalization is an early event that is subsequently inhibited when the microcolonies develop into a more diffuse layer on endothelial cells (Eugene et al., 2002). The formation of the internalization vacuole for endothelial cell invasion has been extensively investigated. Type IV pili binding to CD147/Basigin triggers tyrosine phosphorylation through c-Src and focal adhesion kinase, and EGFR is activated (Slanina et al., 2010, Slanina et al., 2012, Slanina et al., 2014), and acid sphingomyelinase increases ceramide levels on the plasma membrane, forming platforms for the recruitment of host receptors (Simonis et al., 2014; Fohmann et al., 2023). Ezrin, moesin, CD44, ICAM-1, and the tyrosine kinase receptor ErbB2 are recruited. Rho and Cdc42 GTPases orchestrate actin polymerization, and microvilli-like structures are formed (Hoffmann et al., 2001; Eugene et al., 2002). Opc-positive meningococcal lineages have been demonstrated to be more efficiently internalized. This is related to the Opc binding to fibronectin and vitronectin in the host serum, which act as a bridge for the connection with integrin α5β1 and αvβ3, respectively. Opc-mediated internalization is likely host-specific; in fact, *in vitro*, only in the presence of human serum, and not the routinely used bovine-derived serum, were meningococci internalized into endothelial cells (Unkmeir et al., 2002). Additionally, Opc binds preferentially to sulphated tyrosine residues of activated vitronectin (Sa E Cunha et al., 2010), the positions of which are different between vitronectins from different species. Coherently, Opc was able to bind *in vitro* activated human vitronectin but not bovine or murine vitronectins (Sa E Cunha et al., 2010). Opc-mediated binding to integrins led to the formation of focal adhesions with the recruitment of vinculin, talin, paxillin, and cortactin. The last binds and activates the Arp2/3 complex, a central organizer of actin filament structure crucial for Opc-mediated internalization. In fact, a cortactin mutation interrupting the binding to the Arp2/3 complex has been demonstrated to reduce the number of internalized bacteria into endothelial cells (Slanina et al., 2012).

It is worth noting that the internalization vacuole formed during gonococcal infection is quite different from that formed during meningococcal infection. *N. meningitidis* triggers the formation of microvilli-like protrusions (Eugene et al., 2002) while *N. gonorrhoeae* induces membrane ruffles and lamellipodia (Edwards et al., 2000). This discrepancy may contribute to the differences in the intracellular fate of the two pathogens, likely due to the distinct target cells and virulence factors possessed by the bacteria. For instance, Opc is possessed only by the meningococcus, and Opa proteins have different affinities for CEACAM members, with meningococcal Opa primarily targeting CEACAM1 and gonococcal Opa proteins binding to CEACAM1, CEACAM5, CEACAM6, and CEACAM3 on epithelia, neutrophils, and lymphocytes (Sadarangani et al., 2011; Martin et al., 2016).

*N. meningitidis* binds to the hetero-oligomeric complexes formed by CD147 and the G-protein-coupled receptor (GPCR) β2-adrenergic receptor on endothelial cells through Type IV pili (Coureuil et al., 2010). This led to a biased activation of β-arrestin, which regulates GPCRs (Coureuil et al., 2010), promoting endocytosis. Disruption or delocalization of ZO-1, occludin, and claudin-5 has been observed *in vitro* in endothelial cells infected with *N. meningitidis* (Martins Gomes et al., 2019). The β-arrestin signaling has been associated with junctional protein depletion; in fact, GPCRs bind to PDZ-domain containing proteins such as ZO-1, affecting the localization and stability of junctional proteins (Dunn and Ferguson, 2015; González-Mariscal et al., 2018). Moreover, GPCR signaling has been shown to transactivate different metalloproteinases (MMPs) (Lin, 2025), and MMP-8 has been found critical in occludin cleavage upon meningococcal infection in endothelial cells (Schubert-Unkmeir et al., 2010). Mislocalization and degradation of these proteins have been associated with a leakage of the endothelial barrier sufficient for the meningococcus to cross it via the paracellular route. However, meningococci enter the brain with a minimal increase in BBB permeability. Moreover, meningococcal meningitis is not associated with the formation of thrombotic lesions (Join-Lambert et al., 2013), making it unlikely that the barrier will be crossed following cell death. Most observations suggestive of barrier loss were made *in vitro*, and although a paracellular route is possible and likely to occur, the internalization of meningococci in endothelial cells has been clearly demonstrated (Eugene et al., 2002; Doulet et al., 2006; Nikulin et al., 2006; Dupin et al., 2012; Martins Gomes et al., 2019). The *N. meningitidis* MC58 strain was shown to be internalized into the endothelial cells and to cross the barrier within 24 hours without disruption of junctional proteins or alteration in TEER, indicating a transcellular crossing (Endres et al., 2022). Alterations in barrier functions were instead observed by the authors later in the infection (Endres et al., 2022). In agreement, genes involved in barrier permeability, as well as genes involved in cell surface rearrangements and endocytosis, were found to be differentially regulated in endothelial cells infected with the meningococcus (Káňová et al., 2019).

Type IV pili interaction with CD147 on endothelial cells has also been demonstrated to induce an increase in sphingosine 1-phosphate receptor 2 (S1PR2) and the activation of the S1P-S1PR2-EGFR axis, leading to bacterial uptake (Fohmann et al., 2023). S1P is a signaling sphingolipid produced by the phosphorylation of sphingosine by two sphingosine kinases, SphK1 and SphK2 (Maceyka et al., 2012). Meningococcal infection induces sphingosine phosphorylation through SphK1 in a mechanism strictly dependent on pili-mediated interaction with the cell (Fohmann et al., 2023). Consequently, a continuous release of S1P is observed (Fohmann et al., 2023). The latter binds to the receptors S1PR1-5. S1PR1, S1PR2, and S1PR3 are ubiquitous, while S1PR4 and S1PR5 expression is restricted to hematopoietic cells (Sun et al., 2024). Notably, while S1PR2 is upregulated and induces the internalization of meningococci, the activation of S1PR1 or S1PR3 reduces the number of intracellular bacteria (Fohmann et al., 2023). S1P is a regulator of BBB integrity. The interaction of S1P with S1PR1 improves barrier function, while interaction with S1PR2 causes barrier leakage. The balance between S1PR1–3 and S1PR2 signaling is critical for both paracellular and transcellular integrity of the BBB (Garcia et al., 2001; Prager et al., 2015; Wiltshire et al., 2016). In particular, S1PR1 signaling promotes the assembly and maintenance of adherent junctions (Anwar and Mehta, 2020) while S1PR2 signaling disrupts adherent junctions via Rho/ROCK, which can also induce the phosphorylation of occludin and claudin-5, affecting tight junctions (Hirase et al., 2001; Sanchez et al., 2007).

Additionally, internalized bacteria can amplify the inflammatory response, again affecting junctional proteins. Intracellular bacteria have been shown to induce activation of BAD, BAX, caspase-3, and AMPK (Schubert-Unkmeir et al., 2007). Alteration of ion homeostasis is also observed, together with the induction of HIF1 signaling and oxidative stress (Martins Gomes et al., 2019). Caspase-3 activation can have a strong impact on barrier integrity. In fact, it has been demonstrated that caspase-3 leads to ZO-1 and claudin-5 disruption in endothelial cells even in the absence of apoptotic death, indicating a non-apoptotic role in barrier regulation (Zehendner et al., 2011; Yu et al., 2016).

**Figure 2:**
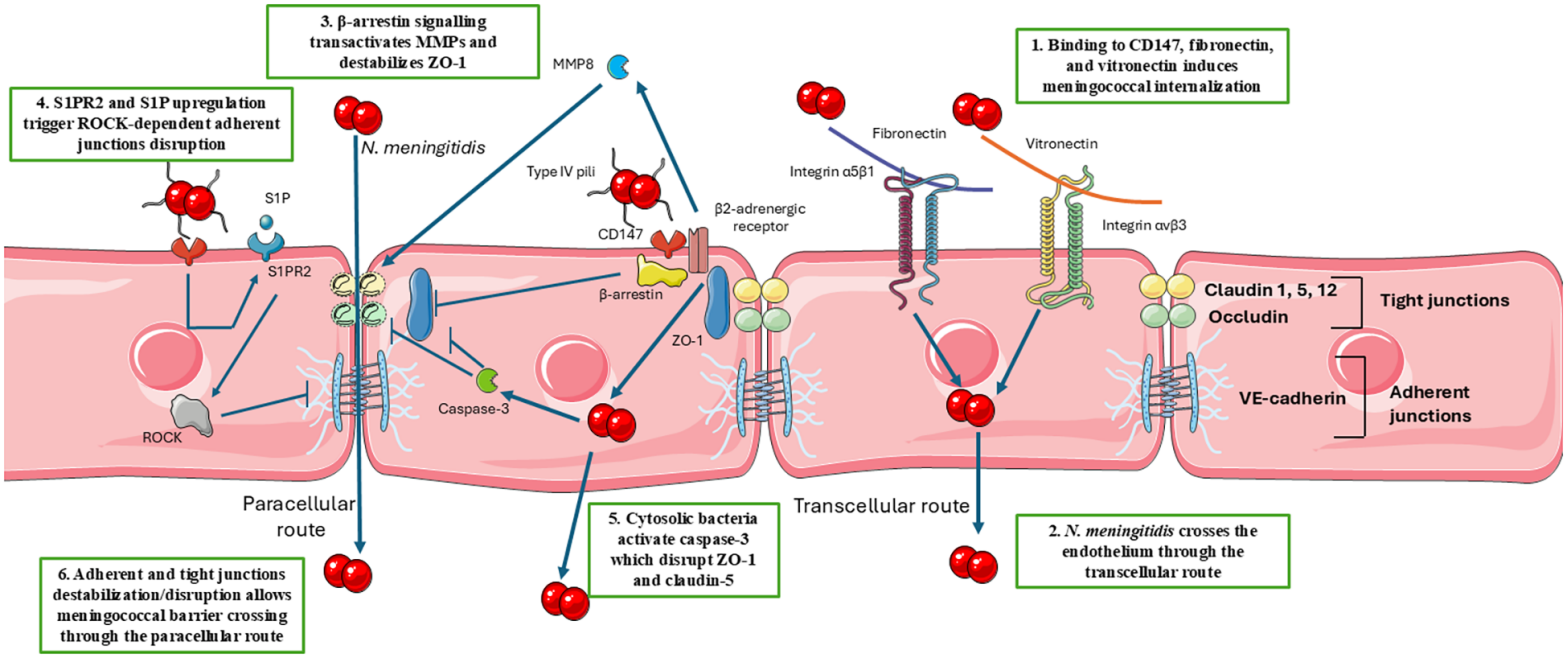
Proposed mechanism of meningococcal crossing of the blood-brain barrier (BBB). Step 1 - In endothelial cells, type IV pili of meningococcus bind to CD147, and Opc binds to fibronectin and vitronectin, inducing bacterial internalization. Step 2 – Internalized meningococci can cross the barrier through the transcellular route. Step 3 - Type IV pili binding to the CD147 and the GPCR β-2 adrenergic receptor complex induces β-arrestin signaling, promoting GPCR interference with ZO-1 and MMP8 production. The latter is secreted and induces occludin cleavage. Step 4 - The sphingosine 1-phosphate (S1P) is upregulated together with S1PR2, which signals induce adherent junction disruption via ROCK. Step 5 - Intracellular bacteria induce caspase-3 activation, among many proinflammatory mediators, which disrupts claudin-5 and ZO-1. Step 6 - The combination of extracellular and intracellular signaling finally interrupts barrier integrity, allowing the paracellular route of infection. Adapted from Servier Medical Art (https://smart.servier.com), licensed under CC BY 4.0 (https://creativecommons.org/licenses/by/4.0/).

Thus, considering also the natural inhibition of endocytosis in the BBB barrier, it is possible that early in infection, only a few meningococci can cross the barrier through the transcellular route. Later in the infection, the combination of signals and pathways induced for bacterial internalization may promote a localized loss of barrier function, allowing the paracellular route to occur, making bacterial internalization a requisite step for further invasion. Although the barrier function is maintained after the bacteria cross it, this step is essential in meningococcal pathogenesis. In fact, this allows reaching the CNS before an intense inflammatory response is elicited. Once in this microenvironment, the replication of the meningococcus in the CFS promotes the intense inflammation leading to fulminant meningitis. Hence, BBB crossing prevention, blocking *N. meningitidis* dissemination to the CNS, could have a major impact on disease outcomes and its sequelae. The proposed mechanisms for the BBB crossing by the meningococcus are illustrated in **Figure 2**.

## 6 Metabolic cross-talk between *N. meningitidis* and the host cell

### 6.1 Carbon and energy metabolism

Besides structures devoted to adherence and invasion in the host, metabolic adaptation is a prerequisite for meningococcal intracellular survival. Meningococci use restricted carbon sources: glucose, lactate, L-glutamate, and pyruvate, and it has to adapt to different host microenvironments (Smith et al., 2007; Antunes et al., 2015; Armstrong, 2015). Glucose and L-glutamate are present primarily in the blood and cerebrospinal fluid, while lactate is the main carbon source in the nasopharynx. Within phagocytic cells, however, pyruvate and lactate are thought to be the main carbon sources, and glutamate metabolism plays a key role in the intracellular survival of meningococci.

Lactate is preferentially used by the meningococcus as a carbon source. LctP efficiently transports lactate into *N. meningitidis* (Exley et al., 2005), which metabolizes the lactate by at least three lactate dehydrogenases: two membrane-bound flavin mononucleotide-containing lactate dehydrogenases, LldD and LdhD, which catalyze the oxidation of *L*-lactate and *D*-lactate, respectively, and a soluble NAD^+^-dependent *D*-lactate dehydrogenase, LdhA (Erwin and Gotschlich, 1993, Erwin and Gotschlich, 1996; Smith et al., 2001; Atack et al., 2014).

Lactate not only has a nutritional role, providing reducing power to the electron transport chain when it is oxidized to pyruvate, which is then channeled into the tricarboxylic acid (TCA) cycle, which is fully active when lactate is used as a carbon source (Jyssum, 1960; Holten, 1974; Smith et al., 2001), but is also involved in virulence. Indeed, sialic acid production, used for capsule biosynthesis and LOS modification, is fueled by lactate. Intermediates of lactate catabolism are directed into the sialylation pathway, aiding capsule biosynthesis for survival in the intracellular environment. Meningococci mutated for the lactate permease gene *lctP* are deficient in sialic acid alteration of the outer membrane and more vulnerable to phagocytic destruction (Llibre et al., 2021; Joshi and Saroj, 2023).

Moreover, this LctP permease seems to be essential for the nasopharyngeal colonization (Exley et al., 2005). Notably, a key regulator of intracellular lactate availability is CD147. The latter acts as a chaperone for monocarboxylate transporter 1 (MCT1) and MCT4, facilitates their proper expression at the cell surface, and prevents their degradation. MCT1 and MCT4 mediate export and import of lactate, but without CD147 association, they accumulate in the perinuclear region and are finally degraded by the proteasome (Kirk et al., 2000; Kendrick et al., 2017). In the brain, lactate produced by astrocytes is secreted through MCT4 in association with CD147, and neurons absorb it through MCT2; CD147 is also essential for the correct membrane localization of this transporter (Kirk et al., 2000; Muramatsu, 2016). Thus, although it has to be demonstrated, Type IV pili binding to CD147 may alter intracellular lactate availability, providing the meningococcus with a local nutritional advantage.

The carbon source available may influence the intracellular survival of meningococci, depending also on the cell type infected. In fact, it has been shown that lactate, pyruvate, and acetate decrease adherence to and invasion of epithelial cells, probably because of the alteration of capsule biosynthesis and adhesin expression. Meningococcal growth in the presence of lactate and pyruvate, in particular, has been shown to decrease expression of genes for capsule synthesis together with *Opa*, *pilE*, *pilT*, and *pilX* (Kanojiya et al., 2022b). On the contrary, lactate, pyruvate, and cysteine enhanced intracellular survival/replication inside phagocytic cells such as macrophages (Kanojiya et al., 2022b).

In contrast, glucose does not appear to be a primary carbon source used by meningococci in the intracellular environment. *N. meningitidis* lacks the phosphotransferase system to efficiently transport glucose and uses an ion symport permease, GluP (also called GlcP), to transport glucose (Derkaoui et al., 2016). In addition, there is no evidence that meningococcus can utilize glucose-6-phosphate, which is an available carbon source in the intracellular environment, as other pathogenic bacteria do (Maloney et al., 1990). In meningococci, glucose is metabolized largely through the Entner-Doudoroff pathway, which generates relatively small amounts of energy (Holten, 1974). Furthermore, at neutral pH, glucose catabolism results in the accumulation of acetate, which is not further catabolized until glucose is exhausted. Indeed, growth on glucose markedly reduces the levels of TCA cycle enzymes in these bacteria (Hebeler and Morse, 1976; Morse et al., 1986; Antunes et al., 2015). The activity of the TCA cycle may be supported by the TCA cycle intermediates succinate, fumarate, malate, and α-ketoglutarate (Weiss, 1970; Holten, 1974).

A notable trait distinguishing *N. meningitidis* from commensal *N. lactamica* is the methylcitrate cycle (MCC). *N. meningitidis* contains a genomic island (*prp*) that enables it to utilize propionic acid by MCC under nutrient-poor growth conditions and overcome propionic acid toxicity (Catenazzi et al., 2014). Using the MCC cycle, propionate is converted to pyruvate and succinate, which can enter the TCA cycle. The *prp* island, comprising *prpB*, *prpC*, *acnD*, *prpF*, and *ackA2* genes, is absent in *N. lactamica*, and this would confer a selective advantage to *N. meningitidis* over *N. lactamica* in young adults (Catenazzi et al., 2014). Indeed, *N. lactamica* and *N. meningitidis* have age-related colonization patterns (Gold et al., 1978; Cartwright et al., 1987; Bennett et al., 2005), and it has been observed that in the pharynx of young adults the increase of meningococcal carriage rate is correlated with an increase in the abundance of bacteria producing propionic acid, an organic acid that is toxic to many microorganisms (Dolan et al., 2018) and can be used instead as an additional carbon source by *N. meningitidis*, but not by *N. lactamica* (Catenazzi et al., 2014). It has been recently shown that the *prp* genomic island is in decay in *N. gonorrhoeae*, and that in several meningococcal lineages the island contains an additional gene, *kbuT* encoding a transporter from the 4-Toluene Sulfonate Uptake Permease (TSUP) family involved in transport of α-ketobutyrate, an α-keto acid particularly abundant in polymorphonuclear neutrophils (PMNs) (Mühling et al., 2003), which can be metabolized through the MCC, raising the hypothesis of a role of MCC during meningococcal host cell infection (Talà et al., 2025).

*N. meningitidis* is generally considered a strictly aerobic bacterium and is typically grown under completely aerobic conditions. However, under oxygen-limiting conditions, it expresses a denitrification pathway that begins with nitrite and ends with nitrous oxide (N_2_O), via nitric oxide (NO) (Rock et al., 2005). Genome and biochemical analyses of pathogenic and commensal species revealed that all *Neisseria* species have a highly conserved nitric oxide reductase (NorB) and nitrite reductase (AniA or NirK), while only *Neisseria mucosa* contained a nitrate reductase (Nar), and only *Neisseria lactamica*, *Neisseria cinerea*, *Neisseria subflava*, *Neisseria flavescens*, and *Neisseria sicca* contained a nitrous oxide reductase (Nos) complex (Barth et al., 2009). Thus, a notable difference between human commensal and pathogenic *Neisseria* species is the lack of a functional Nos system in the pathogenic species *N. gonorrhoeae* and *N. meningitidis*, pointing to a major role of this incomplete denitrification pathway and, possibly, nitrous oxide (N_2_O), a known modulator of N-methyl-D-aspartic acid (NMDA) receptor function (Kashiba et al., 2002), in *N. meningitidis* pathogenicity.

It is also noteworthy that a frameshift mutation abolishes *aniA* expression in a considerable proportion of *N. meningitidis* strains (Ku et al., 2009; Schoen et al., 2014), resulting in differences within the meningococcal population that may impact virulence. Indeed, the denitrification pathway of *N. meningitidis* is strongly involved in detoxification during human host infection. Macrophages produce NO, and NO contributes to host innate immunity both through bactericidal activity and as a signaling molecule. *N. meningitidis* utilizes NorB (nitric oxide reductase) and CycP (cytochrome c’) to detoxify NO, and NorB and to a lesser extent CycP enhanced the survival of *N. meningitidis* in primary human macrophages and nasopharyngeal mucosal organ culture (Stevanin et al., 2005), demonstrating a crucial role of the denitrification pathway during meningococcal passage through the nasopharyngeal barrier and survival in phagocytic cells.

### 6.2 Amino acid metabolism and oxidative stress response

Glutamate metabolism is also critical for meningococcal virulence, linking carbon and nitrogen utilization to the oxidative stress response. In addition to representing an important carbon source, useful to support the activity of the TCA cycle as a direct precursor of α-ketoglutarate (Mallavia and Weiss, 1970; Pagliarulo et al., 2004; Monaco et al., 2006; Colicchio et al., 2009). L-glutamate is also the precursor of glutathione (L-γ-glutamyl-L-cysteinylglycine, GSH). Thus, inside the host, L-glutamate taken from the host cells is critical for meningococcal intracellular fitness, as it is a source of α-ketoglutarate to support the activity of the TCA cycle and protect meningococci from oxidative stress as a precursor of glutathione (Li et al., 2009; Monaco et al., 2006; Schoen et al., 2014; Talà et al., 2008, Talà et al., 2011; Takahashi et al., 2015).

In *N. meningitidis*, L-glutamate metabolism is accomplished by two glutamate dehydrogenase (GDH) activities specific for NADP (NADP-GDH) or NAD (NAD-GDH) that have been known for over fifty years (Holten and Jyssum, 1973). The meningococcal genome has a glutamine synthetase but lacks a functional glutamate synthase and, therefore, a Glutamine Synthetase (GS)/Glutamine Oxoglutarate Aminotransferase (GOGAT) cycle, suggesting a major role for NADP-GDH in ammonia assimilation. Furthermore, old biochemical studies in meningococci and gonococci show that: i. NADP-linked and the NAD-linked activities have different temperature sensitivity and pH optima (eight for NADP-GDH and nine for NAD-GDH); ii. NADP-linked activities are usually higher (generally one order of magnitude) than NAD-linked ones; iii. NAD-linked enzyme is decreased when glucose is added to a chemically defined medium, whereas the activity of the NADP-linked one is increased under the same conditions (Holten, 1973; Holten and Jyssum, 1973). These findings suggest that, unlike other bacteria that use NADP-GDH primarily for ammonia assimilation, *N. meningitidis* can also use NADP-GDH for L-glutamate catabolism to feed the TCA with α-ketoglutarate, depending on the prevailing carbon source in the different host microenvironments (Pagliarulo et al., 2004; Schoen et al., 2014).

In this regard, it can be noted that transcription of the *gdhA* gene initiates from two promoters, one weak, constitutively expressed, called *gdhA* P1, and another, more potent, called *gdhA* P2, transactivated by GdhR, a transcriptional regulator whose DNA binding is inhibited by α-ketoglutarate (Pagliarulo et al., 2004). It is therefore assumed that transcription from *gdhA* P2 serves to enhance *gdhA* expression when the concentration of α-ketoglutarate is low, as during invasive disease, both to enhance the activity of the TCA cycle in the catabolic reaction, and to provide L-glutamate for glutathione biosynthesis in the anabolic reaction. Indeed, in *N. meningitidis* MC58, *gdhA* expression was particularly increased in response to glucose *in vitro*, a condition that decreased the expression of TCA cycle genes (Antunes et al., 2015). Interestingly, *gdhA* transcription from *gdhA* P2 is significantly higher *in vitro* in meningococcal strains belonging to hypervirulent lines compared to those belonging to carrier lines expressing low levels of GhdR (Pagliarulo et al., 2004) as will be discussed below.

In further support of the important role of L-glutamate metabolism in meningococcal virulence, *gdhA* was found to be essential for meningococcal survival in the infant rat model (Sun et al., 2000). Moreover, L-glutamate transport plays a key role during meningococcal host infection. *N. meningitidis* possesses a Na^+^-dependent L-glutamate transporter, GltS, and an ABC-type transporter required for Na^+^-independent L-glutamate transport, GltT (Monaco et al., 2006; Takahashi et al., 2015). The GltT transporter comprises an ATP-binding protein (NMB1966), an inner membrane permease (NMB1965), an outer membrane substrate binding protein (NMB1964), and a periplasm transport protein (NMB1963). Additional components could be an NTP-binding protein (STAS) (NMB1962) and a VacJ-like lipoprotein (NMB1961) (Monaco et al., 2006).

GltT was required for meningococcal survival within phagocytic cells (Talà et al., 2011) and in human whole blood (Li et al., 2009). Furthermore, GltT-defective mutants, but not GltS-defective mutants, showed slightly reduced survival in HeLa cells (Monaco et al., 2006), and were significantly attenuated in a murine model of meningococcal meningitis (Colicchio et al., 2009) and in systemic murine models of infection (Li et al., 2009; Talà et al., 2011). Interestingly, the GltT-defective mutant also exhibited less adhesive and invasive properties to human bronchial epithelial cells, and transcriptome analysis suggested that genetic inactivation of *gltT* led to remodeling of the outer membrane and surface structures (Li et al., 2009). To explain this finding, it may be noted that GltT, although required for L-glutamate transport, is highly homologous to the VacJ/Yrb ABC transport system, a proposed phospholipid transporter for maintaining lipid asymmetry in the Gram-negative outer membrane (Mla pathway) (Malinverni and Silhavy, 2009), also involved in outer membrane vesicle (OMV) formation (Roier et al., 2016).

Consistent with a role of GltT in meningococcal adhesion/invasion to epithelial and endothelial cells, Takahashi and colleagues found that: i. GltT-defective mutants (NMB1965- and/or NMB1964-defective mutants, respectively) were significantly defective in the internalization into human umbilical vein endothelial cells (HBMEC) and the human lung carcinoma epithelial cell line A549; ii. the efficiency of meningococcal invasion of HBMEC decreased under L-glutamate-depleted conditions; iii. Ezrin, a membrane-cytoskeleton linker, accumulated beneath colonies of the wild-type *N. meningitidis* strain but not of the GltT-defective mutant (Takahashi et al., 2011). These findings suggest that L-glutamate influx involving the GltT ABC transport system serves as a cue for *N. meningitidis* internalization into HBMEC (Takahashi et al., 2011), and it was proposed that meningococcal internalization into HBMEC might be induced by the reduced environmental glutamate concentration upon infection (Takahashi et al., 2015). Moreover, evidence was provided that the amount of glutathione within the GltT-defective mutant was much lower than that within the wild type strain only upon HBMEC infection and was correlated with intracellular survival (Takahashi et al., 2015), confirming previous findings with phagocytic cells (Talà et al., 2011). Overall, these results suggest that L-glutamate plays an important role in the intracellular phase of meningococcal cell infection, acting through different mechanisms.

Although there is genetic and biochemical evidence that *N. meningitidis* is able to synthesize all proteinogenic amino acids, its growth is known to be greatly stimulated by L-glutamate, L-arginine, glycine, L-serine and L-cysteine (or L-cystine) supplement (Jyssum, 1959; Wesley Catlin, 1973; Port et al., 1984; Leighton et al., 2001). How meningococcus obtains L-cysteine ​​is crucial to understanding its survival mechanisms in host cells, as cysteine ​​is used for protein and glutathione synthesis, as well as being the main sulfur source for a variety of other molecules, such as biotin, coenzyme A, lipoic acid, and other cysteine ​​derivatives important for protection from oxidative stress. Although the meningococcus is able to synthesize L-cysteine ​​using sulfate (a property that was lost in *N. gonorrhoeae*) or thiosulfate as a sulfur source (Hicks and Mullholland, 2018), cysteine ​​uptake by the meningococcal cysteine ​​transport system (CTS) was crucial for the survival/persistence of *N. meningitidis* in HBMECs (Takahashi et al., 2018). Furthermore, many *N. meningitidis* strains show L-cysteine auxotrophy (Takahashi et al., 2004; Baart et al., 2007; van de Waterbeemd et al., 2013; Ampattu et al., 2017). Evidence is provided that the CTS is fundamental under low L-cysteine concentration, as in human epithelial and endothelial cells, where the intracellular L-cysteine concentration is very low at approximately 100 μM (Alkhuder et al., 2009). After entering the bloodstream, meningococcus encounters a low concentration of L-cysteine, approximately 30–60 μM, in human blood plasma and, after crossing the blood–brain barrier, an even lower concentration of L-cysteine, less than 1 μM, in human cerebrospinal fluid (CFS) (Lindholm et al., 1989; Su et al., 2015; Afzal et al., 2016).

Thus, as well as the L-glutamate uptake and metabolism, the L-cysteine uptake and metabolism play a fundamental role in the glutathione metabolism and γ-glutamyl cycle during meningococcal infection. It is also interesting to note that many of the amino acids that stimulate meningococcal growth, namely, L-glutamate, glycine, L-serine, and L-cysteine (Wesley Catlin, 1973), are involved in glutathione metabolism and the γ-glutamyl cycle. Glutathione is synthesized from the amino acids L-glutamate, L-cysteine, and L-glycine; glycine and L-cysteine are both derived from L-serine, which is generated from 3-phosphoglycerate; glutathione can be catabolized to yield L-cysteine via gamma-glutamyl-aminopeptidase and aminopeptidase N. In turn, L-cysteine can be converted into glutathione via GshA and GshB, yielding a functional γ-glutamyl cycle. The importance of a functional γ-glutamyl cycle for IMD is supported by the evidence that meningococcal gamma-glutamyl-aminopeptidase is essential for growth of *N. meningitidis* in the cysteine-deficient environments, such as the CSF (Takahashi et al., 2004).

### 6.3 Iron and other transition metals

In addition to carbon, sulfur, and nitrogen sources, *N. meningitidis* requires essential elements such as zinc and iron to survive. Intracellular replication has been found to be supported by TonB-dependent iron acquisition from a host source, such as ferritin, the degradation of which is promoted by the pathogen (Larson et al., 2002, Larson et al., 2004). The acquisition of these and other transition metals is essential for the survival of the pathogen in host tissues, and during bacterial infection, a battle ensues between the pathogen and the host: the host responds to the pathogen by limiting the availability of metals, as part of an innate immunity strategy called nutritional immunity and the pathogen attempts to counter this maneuver by all means. One strategy of bacterial pathogens is hijacking abundant host iron-binding proteins. Meningococcus primarily utilizes hemoglobin, transferrin, and lactoferrin as a source of iron, via specific outer membrane systems such as HmbR/HupAB for hemoglobin/heme and TbpA/TbpB and LbpA/LbpB for transferrin and lactoferrin, respectively. The specific source used varies by niche, with lactoferrin dominating in the nasopharynx and also in cerebrospinal fluid during meningitis (lactoferrin crosses the blood-brain barrier during inflammation), and transferrin and hemoglobin dominating in the bloodstream (Criado et al., 1993; Perkins-Balding et al., 2004; Cornelissen and Hollander, 2011a; Neumann et al., 2017).

The iron uptake systems of *N. meningitidis* have been extensively studied and reviewed and will not be discussed here, except for some aspects that have relevance to the interaction between meningococcus and human host cells. An important aspect is the host selectivity and substrate specificity of these systems. HmbR and HpuAB enable meningococci to utilize heme-containing proteins as a source of heme. HmbR scavenges heme from hemoglobin, most efficiently from human hemoglobin (Perkins-Balding et al., 2004). HpuAB utilizes hemoglobin-haptoglobin more efficiently than hemoglobin itself as an iron source and shows a preference for certain forms of the hemoglobin-haptoglobin complex, the gene encoding the haptoglobin α chain being polymorphic in humans with three possible genotypes (Rohde and Dyer, 2004; Cornelissen and Hollander, 2011a) Most invasive strains express HmbR alone or both heme uptake systems, such as those belonging to the cc11, while strains expressing only HpuAB are mostly found among carriage strains (Tauseef et al., 2011). In many invasive strains, including the reference strain MC58, *hpuAB* expression was lost as a consequence of either complete deletion or replacement by an insertion element. Moreover, when present, the expression of *hmbR* and *hpuAB* was subject to ON/OFF phase variation via reversible frameshift mutation in homopolymeric repeats (Lewis et al., 1999). The periplasmic heme-binding protein and the inner membrane heme transporter have not yet been identified, while within the cytoplasm, heme is degraded by heme oxygenase (HemO), resulting in the release of iron (Zhu et al., 2000).

In contrast to HmbR, which preferentially but not exclusively recognizes human hemoglobin, TbpA/TbpB and LbpA/LbpB recognize exclusively human transferrin and lactoferrin, respectively, and this selectivity strongly contributes to the narrow host spectrum of the meningococcus (Perkins-Balding et al., 2004). The outer membrane protein TbpA and the surface lipoprotein TbpB work together to extract iron from human transferrin, with TbpA forming the channel for iron transport across the membrane and TbpB acting as an initial binding partner that enhances iron release and transferrin removal. TbpB specifically recognizes the iron-bound form of transferrin, helping to initiate the iron acquisition process by destabilizing the transferrin molecule (Perkins-Balding et al., 2004; Cornelissen and Hollander, 2011a). TbpA and TbpB show considerable variation, particularly TbpB, whose molecular mass ranges from 68 to 85 kDa in different meningococcal isolates (Perkins-Balding et al., 2004). A similar model involving the outer membrane protein LbpA and the surface lipoprotein LbpB works together to extract iron from human lactoferrin (Perkins-Balding et al., 2004; Cornelissen and Hollander, 2011a) As well as *hmbR* and *hpuAB*, *tbpB* and *lbpB* are subject to phase variation. After transport across the outer membrane, iron recovered from human transferrin or lactoferrin is bound by the periplasmic protein FbpA and directed to the inner membrane transporter FbpBC (Adhikari et al., 1996). Most of the heme and iron outer membrane transport systems require energy provided by the ExbB-ExbD-TonB system (Stojiljkovic and Srinivasan, 1997), although TonB-independent iron transport processes were reported in *N. gonorrhoeae* (Zola et al., 2010).

In addition to these sophisticated systems for extracting iron from host proteins, the meningococcus can also obtain iron through other systems. *N. meningitidis* does not produce siderophores (Archibald and DeVoe, 1980) but instead hijacks catecholate-type siderophores secreted by other bacteria, with broad specificity (Cornelissen, 2018). The crystal structure of meningococcal FetA indicates that this outer membrane protein binds to and perhaps directly internalizes ferric iron, consistent with its broad specificity (Saleem et al., 2012; Cornelissen, 2018). Other components of this siderophore uptake system are a periplasmic binding protein, FetB, and an ABC transport system, FetCDEF (Cornelissen, 2018). The *fetA* gene expression is subject to phase variation.

In addition to iron-loaded xenosiderophores, iron-complexing compounds like citrate and pyrophosphate have been shown to support *N. meningitidis* growth *in vitro* (Archibald and DeVoe, 1980; Biville et al., 2014). Furthermore, it was shown that iron in compounds such as ferrioxamine B, ferrichrome, ferritin, Imferon, cytochrome c, FePO_4_, and [Fe(OH)3]n was not readily available to *N. meningitidis*, but the addition of some of these iron-complexing substances (e.g., citrate and pyrophosphate) in iron-free form made many biologically important iron compounds that are normally inaccessible to the meningococci readily available (Archibald and DeVoe, 1980).

The ability of meningococcus to recover iron from ferritin is of particular interest for understanding the intracellular phase of infection. Non-heme iron in humans is located intracellularly in ferritin, but, despite this large iron reserve, most pathogens are unable to scavenge iron from ferritin. Interestingly, it has been shown that, during infection, *N. meningitidis* can trigger rapid redistribution and degradation of cytosolic ferritin within infected epithelial cells and that cytosolic ferritin is aggregated and recruited to intracellular meningococci (Larson et al., 2004). This finding indicates that ferritin is a major source of iron for meningococci during cellular infection, a hypothesis supported by evidence that: i. supplementing infected epithelial cells with ascorbic acid abolished ferritin redistribution and degradation and prevented intracellular meningococcal replication; ii. the lysosomal protease inhibitor leupeptin slowed ferritin turnover and also delayed meningococcal replication (Larson et al., 2004). Furthermore, meningococcal infection has been shown to subvert iron homeostasis in infected cells by interfering with transferrin uptake by infected cells (Bonnah et al., 2004), which, consequently, displays a transcriptional profile indicative of iron deficiency following meningococcal infection (Bonnah et al., 2004).

As a nutritional immunity strategy, the human host sequesters, in addition to iron, other transition metals essential for bacterial multiplication, including zinc and manganese, in storage proteins. The function of calprotectin is to sequester zinc and manganese. This protein is secreted by neutrophils, monocytes/macrophages, and other cell types (such as epithelium) during pathogen infections, but it is also present in the intracellular milieu (Zhou et al., 2023). Pathogenic *Neisseria* species encode two outer membrane transporters for Zn import, TdfH and TdfJ, originally discovered in *N. gonorrhoeae* as the TonB-dependent family (Tdf) of outer-membrane receptor proteins (Turner et al., 2001; Cornelissen and Hollander, 2011a) and subsequently characterized in *N. meningitidis* (Stork et al., 2010, Stork et al., 2013; Kumar et al., 2012; Calmettes et al., 2015). In addition to binding zinc, TdfH has been shown to bind calprotectin and deliver zinc into the neisserial cell (Jean et al., 2016), and was therefore renamed CbpA, for calprotectin-binding protein A (Stork et al., 2013). In contrast, TdfJ, similar to TdfH, allows bacteria to accumulate zinc, but in this case, zinc uptake is not allowed if zinc is associated with calprotectin (Jean et al., 2016), and was therefore renamed ZnuD (Kumar et al., 2012; Calmettes et al., 2015). The meningococcus overexpresses CbpA when zinc concentration is low, while the concentration of Mn^2+^ does not induce its overexpression. However, like Zn^2+^, the loading of calprotectin with Mn^2+^ stimulates its binding to CbpA-expressing meningococci (Stork et al., 2013). In addition to iron uptake, after crossing the outer membrane, zinc uptake is facilitated by a periplasmic binding protein and a homologous membrane permease system ZnuABC (also called MntABC in the literature), and mutants unable to produce any of these proteins are defective in intracellular survival (Lim et al., 2008).

Intriguingly, two other Tdf proteins are present in pathogenic but in none of the commensal *Neisseria* species, TdfF and TdfG (Marri et al., 2010), suggesting a key role in virulence. Indeed, Hagen and Cornelissen (2006) found that TdfF expression was repressed by iron in *N. gonorrhoeae*, that a *tdfF*-defective mutant was impaired for growth within epithelial cells, and that this growth defect was suppressed by iron supplementation, suggesting a key role for TdfF in iron transport within epithelial cells. In contrast, TbpA, FetA, and LbpA, and the putative transporters TdfG, TdfH, and TdfJ were not required for intracellular survival of gonococci (Hagen and Cornelissen, 2006). A ligand for TdfF has not yet been identified, but, as mentioned above, among the possible candidates for iron sources in the intracellular environment (ferritin, iron chelated to organic acids or to glutathione), only ferritin has been shown to contribute to the intracellular survival of *N. meningitidis* (Larson et al., 2004), indicating this molecule as a possible ligand for TdfF.

In summary, accumulating evidence points to metabolism as a key determinant of meningococcal colonization and invasion, with important differences between hypervirulent and carrier strains (**Table 2**). There is well-established evidence that meningococci utilize limited compounds, glucose,

**Table 2:** Selection of genetic traits that distinguish hypervirulent and carriage meningococcal lineages and strains.

Biological function category	Gene	Function of encoded gene product(s)	Meningococcal hyperinvasive or carriage strain (strain, clonal complex, sequence typing)	References
Virulence factors	*siaB, siaC*	Polysaccharide capsule biosynthesis	Invasive: High expression (MC58, cc32, ST32)	(Joseph et al., 2010, Joseph et al., 2011) (Moreno et al., 2015; Ampattu et al., 2017; Neri et al., 2019; Olof et al., 2023)
			Carriage: expression low (α710, cc136, ST-136) (α522, cc-35, ST-35) or null (cc198; cc1136; and cc53 isolates)	
	*pglI*	Glycosyltransferase	Invasive: High expression (cc11; cc32)	(Mullally et al., 2021)
			Carriage: absent (cc53)	
	*tps loci*	-System 1 (HrpA/HrpB): adhesion to epithelial cells, biofilm formation, vacuolar escape, intracellular movement through dynein binding -System 2: Not Addressed -System 3: Not Addressed	Invasive: overrepresentation of system 2 and system 3 (cc8, cc11, cc32, cc41/44, cc269); System 1 *tpsC* cassettes and IORFs frequently present (cc11; cc32)	(van Ulsen et al., 2008; Mullally et al., 2021)
			Carriage: systems 2 and 3 less represented (cc22; cc60; cc162; cc53) Absence of *tpsC* cassettes and IORFs (cc53)	
	*igA*	IgA cleavage, LAMP1 cleavage, Nf-κB activity regulation when a nuclear localization signal (NLS) is present	Invasive: present a nuclear localization signal (NLS) (ST-11)	(Besbes et al., 2015)
			Carriage: NLS in IgA protease is absent (non-ST-11 isolates)	
	*hmbR and hpuAB*	Scavenger of iron from haemoglobin (HmbR and, less efficiently, HpuAB) and hemoglobin-haptoglobin complex (HpuAB)	Invasive: high prevalence of only *hmbR* (ST-41/44, ST-18, ST-32, ST-269) or both *hmbR* and *hpuAB* locus (cc5; cc8; cc11)	(Tauseef et al., 2011)
			Carriage: high prevalence of only *hpuAB* locus (ST-174; ST-106)	
	*kdtA, lpxC, lpxA, lpxD, lpxB, lgtF, lgtB, rfaC*	LOS synthesis	Invasive: High expression in blood (MC58, cc32, ST-32)	(Ampattu et al., 2017)
			Carriage: Low expression in blood (α522, cc35, ST-35)	
Metabolism	*gdhA* *gdhR*	NADP-dependent glutamate dehydrogenase (NADP-GDH) and its transcriptional regulator GdhR	Invasive: High expression of *gdhA* (ST-32; ST-4)	(Pagliarulo et al., 2004)
			Carriage: low expression of *gdhR* and *gdhA*	
	*argH*, *aroA*, *aroB*, *ilvC*, *gdhA*	Amino acid transport and metabolism	Invasive: High expression (MC58, cc32, ST32)	(Joseph et al., 2010)
			Carriage: Low expression (α710, cc136, ST-136)	
	*atpA*, *atpD*, *atpG*	ATP synthase subunits	Invasive: High expression (MC58, cc32, ST32)	(Joseph et al., 2010)
			Carriage: Low expression (α710, cc136, ST-136)	
	*nqrB, nqrC, nqrD*	Na^+^-translocating NADH-quinone reductase subunits	Invasive: High expression (MC58, cc32, ST32)	(Joseph et al., 2010)
			Carriage: Low expression (α710, cc136, ST-136)	
	*adk*	Adenylate kinase	Significant difference in allelic frequencies between carriage and invasive strains (invasive ST-11; ST-41/44; carriage ST-23; ST-32)	(Yazdankhah et al., 2004)
	*aroE*	Shikimate dehydrogenase	Significant difference in allelic frequencies between carriage and invasive strains (invasive ST-11; ST-41/44; carriage ST-23; ST-32)	(Yazdankhah et al., 2004)
	*fumC*	Fumarate hydratase	Significant difference in allelic frequencies between carriage and invasive strains (invasive ST-11; ST-41/44; carriage ST-23; ST-32	(Yazdankhah et al., 2004)
	*gdh*	Glucose-6-phosphate 1-dehydrogenase	Significant difference in allelic frequencies between carriage and invasive strains (invasive ST-11; ST-41/44; carriage ST-23; ST-32	(Yazdankhah et al., 2004)
	*pdhC*	Pyruvate dehydrogenase subunit E1	Significant difference in allelic frequencies between carriage and invasive strains (invasive ST-11; ST-41/44; carriage ST-23; ST-32	(Yazdankhah et al., 2004)
	*pgm*	Phosphoglucomutase	Significant difference in allelic frequencies between carriage and invasive strains (invasive ST-11; ST-41/44; carriage ST-23; ST-32	(Yazdankhah et al., 2004)
	*gpxA*	Glutathione peroxidase	Invasive: High expression (cc11; cc32)	(Mullally et al., 2021)
			Carriage: absent (cc53)	
	*glmU*	N-acetylglucosamine 1 phosphate (GlcNAc 1 P) uridyltransferase	Invasive: high prevalence of S373C single-nucleotide polymorphism (SNP) (ST-41/44; ST-11)	(Eriksson et al., 2023)
			Carriage: low prevalence of the S373C SNP (ST-32; ST-35; ST-198)	
Genome stability, gene regulation and gene expression machinery	*NEIS1048*	Phage transposase	Invasive: High prevalence (ST-41/44; ST-11)	(Eriksson et al., 2023)
			Carriage: Low prevalence (ST-32; ST-35; ST-198)	
	*rpoD, rpoE*	Sigma factors	Invasive: Low expression (MC58, cc32, ST32)	(Joseph et al., 2010)
	*rpoD, rpoE* *tuf*	Sigma factors Elongation factor Tu	Carriage: High expression (α710, cc136, ST-136)	(Joseph et al., 2010) (Joseph et al., 2010)
			Invasive: Low expression (MC58, cc32, ST32)	
	*tuf* *clpX*	Elongation factor Tu Clp protease	Carriage: High expression (α710, cc136, ST-136)	(Joseph et al., 2010) (Joseph et al., 2010)
			Invasive: Low expression (MC58, cc32, ST-32)	
	*clpX* *rpsJ, rplD, rplW, rplB, rpsS, rplV, rplP, rpsQ, rplX, rpsN, rplF, rplR, rplE, rpsM*	Clp protease Ribosomal proteins	Carriage: High expression (α710, cc136, ST-136)	(Joseph et al., 2010) (Joseph et al., 2010)
			Invasive: Low expression (MC58, cc32, ST32)	
	*rpsJ, rplD, rplW, rplB, rpsS, rplV, rplP, rpsQ, rplX, rpsN, rplF, rplR, rplE, rpsM* *mutS, mutL*	Ribosomal proteins DNA mismatch repair	Carriage: High expression (α710, cc136, ST-136)	(Joseph et al., 2010) (Colicchio et al., 2006a)
			Invasive: Genetic inactivation of *mutS* by IS1106 insertion; Defective *mutL* alleles by missense mutations (93/4286, cc11, ST-11) (ST-44 isolates)	
	*mutS, mutL* *relA*	DNA mismatch repair Guanosine 3’-(tri)diphosphate 5-’diphosphate ((p)ppGpp) synthetase	Carriage: *mutS* and *mutL* genetically stable	(Colicchio et al., 2006a ), (Ampattu et al., 2017)
			Invasive: High expression in blood (MC58, cc32, ST-32)	
	*relA* *misR*	Guanosine 3’-(tri)diphosphate 5-’diphosphate ((p)ppGpp) synthetase PhoP-family response regulator	Carriage: Low expression in blood (α522, cc35, ST-35)	(Ampattu et al., 2017) (Ampattu et al., 2017)
			Invasive: High expression in blood (MC58, cc32, ST-32)	
	*misR* *rpoE*	PhoP-family response regulator Alternative sigma factor σ^E^	Carriage: Low expression in blood (α522, cc35, ST-35)	(Ampattu et al., 2017) (Ampattu et al., 2017)
			Invasive: High expression in blood (MC58, cc32, ST-32)	
	*rpoE* *fur*	Alternative sigma factor σ^E^ Fur, major transcriptional regulator of iron homeostasis	Carriage: Low expression in blood (α522, cc35, ST-35)	(Ampattu et al., 2017) (Ampattu et al., 2017)
			Invasive: High expression in blood (MC58, cc32, ST-32)	
	*fur* *fnr*	Fur, major transcriptional regulator of iron homeostasis FNR, master regulator for adaptation to oxygen-limited conditions	Carriage: Low expression in blood (α522, cc35, ST-35)	(Ampattu et al., 2017) (Ampattu et al., 2017)
			Invasive: High expression in blood (MC58, cc32, ST-32)	
	*fnr*	FNR, master regulator for adaptation to oxygen-limited conditions	Carriage: Low expression in blood (α522, cc35, ST-35)	(Ampattu et al., 2017)

lactate, L-glutamate, and pyruvate, as their major carbon sources, and that the relative abundance of these carbon sources is different in the different host microenvironments relevant for meningococcal infection (nasopharynx, blood, cerebrospinal fluid, epithelial cells, endothelial cells, phagocytes) and subject to variation during meningococcal invasive disease. Therefore, the ability of bacteria to exploit and manage the utilization of these carbon sources in host microenvironments is critical for meningococcal colonization and disease. Therefore, not surprisingly, *N. meningitidis* establishes a metabolic cross-talk with the host cell and is able to modulate the expression of virulence genes in response to prevailing carbon sources. Lactate, pyruvate, and acetate reduce epithelial cell adherence and invasion, likely due to altered capsule biosynthesis and adhesin expression. Conversely, lactate, pyruvate, and cysteine ​​enhance intracellular survival/replication within phagocytic cells such as macrophages. In addition to these carbon sources, *N. meningitidis* can utilize propionate, a compound toxic to many bacteria, through the MCC. *N. lactamica* is not able to utilize the toxic propionate, and therefore, the increased colonization of the human nasopharynx by propionic bacteria with age would explain the associated decline in colonization by *N. lactamica* and the subsequent increased likelihood of colonization by the meningococcus. *N. meningitidis* survival in the intracellular environment is favored by the L-glutamate and lactate meningococcal metabolisms as well as the meningococcal capability to detoxify NO. In addition, recently it has been hypothesized that it can use alternative intracellular carbon sources, such as α-ketobutyrate, through the MCC. In the intracellular environment, meningococcal amino acid metabolism promotes bacterial survival because it is involved in protection against oxidative damage. In particular, L-glutamate and L-cysteine metabolism play key roles in glutathione synthesis and metabolism, and the γ-glutamyl cycle ensures the recycling of cysteine/glutamate and so the maintenance of the glutathione pool. Finally, zinc and iron are essential elements for meningococcal survival, so the bacterium develops strategies to acquire them from the host both extracellularly and intracellularly. It is emerging that ferritin most likely represents the main iron source in the intracellular milieu. Of particular interest are the meningococcal Tdf proteins, specialized in iron and zinc acquisition. They substantially contribute to the narrow host specificity of the meningococcus; some of them are found only in pathogenic *Neisseria* spp. and are essential for intracellular survival. Key factors influencing the intracellular/extracellular viability of *N. meningitidis* in different host microenvironments relevant to meningococcal colonization and IMD are summarized in **Figure 3**.

**Figure 3:**
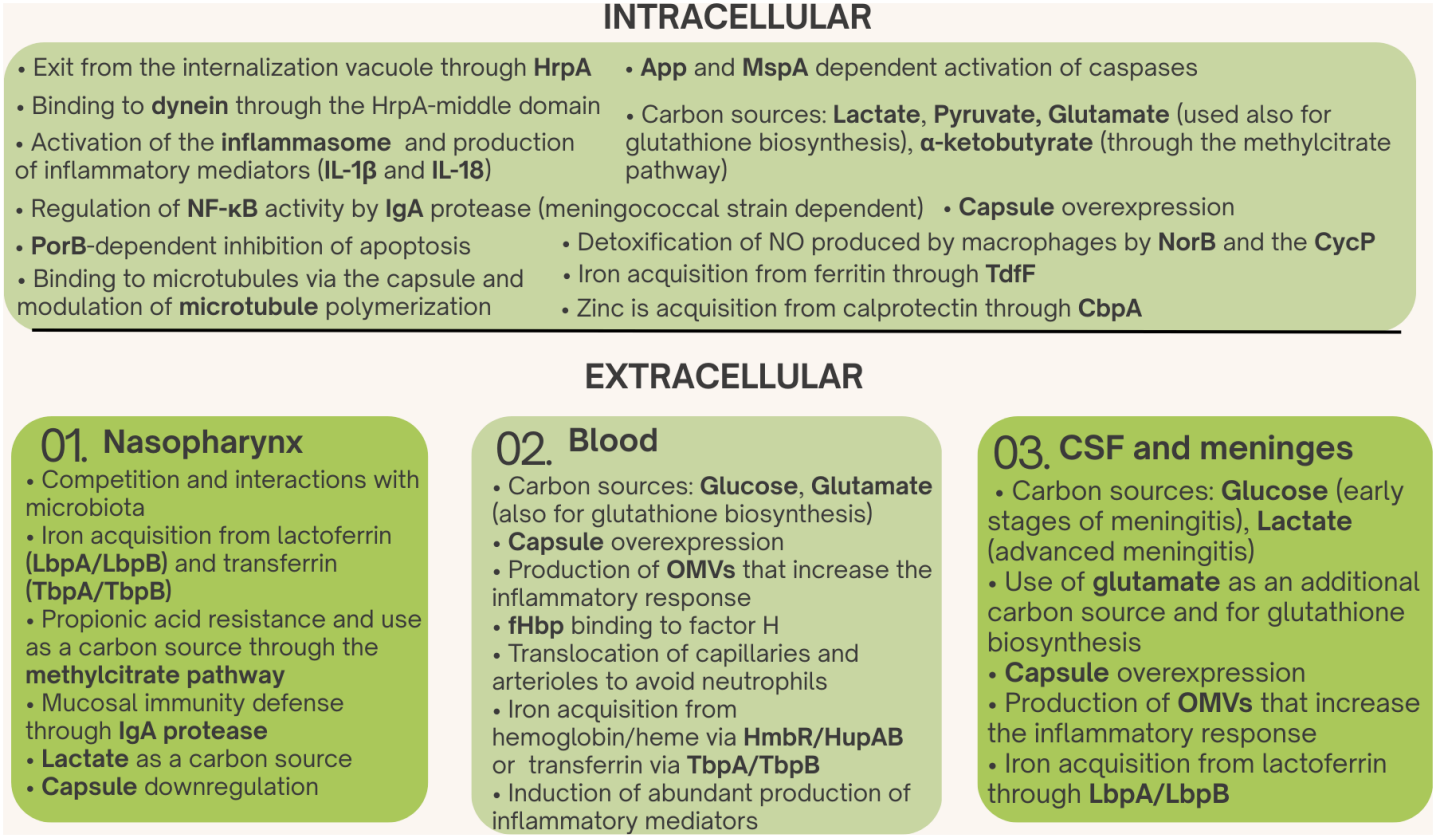
Key factors influencing the intracellular/extracellular viability of *N. meningitidis* in different host microenvironments.

## 7 Variation in gene expression regulation, epigenetic control, and virulence

Since, as mentioned above, most virulence determinants are uniformly distributed in the *N. meningitidis* population with no obvious difference between the clonal complexes mostly associated with IMD and those rarely found in IMD cases, differential gene expression has been proposed as an important determinant of the hyperinvasive phenotype (Joseph et al., 2011; Schoen et al., 2014; Tan et al., 2016; Ampattu et al., 2017; Ren et al., 2017). This indication arises from the results of some fairly recent comparative transcriptome studies, but also from some previous studies on the expression of single genes.

By comparatively analyzing the overall transcriptional profile of the MC58 strain, belonging to the hyperinvasive cc32 (ST-32), with that of the α710 strain, a carrier isolate belonging to the cc136 (ST-136), during adhesion to human nasopharyngeal cells, Joseph et al. (2010) found notable differences in the expression of specific genes mainly involved in meningococcal metabolism. In particular, the 55 most highly expressed genes in the invasive MC58 strain included genes encoding proteins involved in amino acid transport and metabolism (*argH*, *aroA*, *aroB*, *ilvC*, and *gdhA*), genes for ATP synthase subunits (*atpA*, *atpD*, *atpG*), genes involved in sialic acid capsule biosynthesis (*siaB*, *siaC*), and an operon encoding Na^+^-translocating NADH-quinone reductase subunits (*nqrB*, *nqrC*, *nqrD*). In contrast, the 81 genes that were higher expressed in the carriage strain α710 comprised, among the others, genes involved in inorganic ion transport and metabolism and genes encoding two sigma factors (*rpoD* and *rpoE*), the *tuf* genes for the elongation factor Tu, *clpX* (encoding the Clp protease), a *tpsA* gene, and 14 genes encoding ribosomal proteins.

Furthermore, it is interesting to note that, with the exception of *abcZ*, which encodes an ABC transporter, the other six housekeeping genes used to analyze the structure of the meningococcal population by MLST to identify strains belonging to certain ccs, more or less associated with meningococcal disease or carrier status, encode enzymes involved in the main metabolic pathways (Yazdankhah et al., 2004; Maiden, 2006). Specifically, *adk* encodes adenylate kinase, *aroE* encodes shikimate dehydrogenase required for chorismate biosynthesis, *fumC* encodes fumarate hydratase of the tricarboxylic acid cycle (TCA), *gdh* encodes the glucose-6-phosphate 1-dehydrogenase, *pdhC* (*aceE*) encodes the pyruvate dehydrogenase subunit E1, and *pgm* encodes phosphoglucomutase. Although genetic variations at these loci have long been considered neutral, the availability of a significant amount of epidemiological data and the use of mathematical models have allowed us to discover that some combinations of alleles at these loci may be subject to selection and that some combinations in some hypervirulent lines are associated with small gains in fitness for meningococcal transmission (Buckee et al., 2008). These data support the link between metabolic efficiency, increased transmission fitness, and increased incidence of IMD in several clonal complexes as theorized (Stollenwerk et al., 2004; Moxon et al., 2006).

This conclusion was supported by comparative genomic, transcriptomic and phenotypic profiles of meningococcal isolates from disease patients and their household contacts, which led to identification of potentially important metabolic differences between carriage and disease isolates including the sulfate assimilation pathway, in addition to the observation that several carriage isolates had lost their type IV pili and that this loss correlated with reduced induction of tumor necrosis factor alpha (TNF-α) expression when cultured with epithelial cells (Ren et al., 2017).

An interesting study compared the gene expression of the invasive MC58 strain and the α522 carrier strain under *ex vivo* conditions simulating the commensal and virulence compartments to assess the strain-specific impact of gene regulation on meningococcal virulence (Ampattu et al., 2017). The authors found that these strains differed in the expression of over 500 genes under conditions mimicking infection, in spite of indistinguishable *ex vivo* phenotypes. These differences specifically included metabolic genes, including those involved in the incomplete denitrification pathway, information processing genes, as well as genes known to be involved in host damage, and numerous LOS biosynthesis genes. Furthermore, a model-based analysis of transcriptomic differences in human blood suggested differences in metabolic flux in the energy, glutamine, and cysteine metabolic pathways, along with differences in stringent response activation (Ampattu et al., 2017). This computational result was supported by experimental analyses that revealed differences in conditional cysteine and glutamine auxotrophy, as well as a strain- and condition-dependent essentiality of the (p)ppGpp synthase gene *relA* associated with a short non-coding AT-rich repeat element in its promoter region (Ampattu et al., 2017). The conclusion of this study is that some differences between different meningococcal strains in the expression of certain metabolic traits may be cryptic due to “transcriptional buffering”, and can be revealed only under particular stress conditions. This study also highlights the importance of the stringent response in meningococcal virulence and the differences in its activation in different meningococcal strains. This study also shows that the gene differences between the MC58 pathogenic strain and the α522 carrier, in particular in the blood, may be caused by the activation of different sets of regulatory genes, in addition to RelA, including those encoding: i. MisR, a PhoP-family response regulator of the two-component signal transduction system MisR/MisS that regulates various cellular processes in pathogenic *Neisseria*, including LOS structure and modification, iron assimilation, and resistance to host defenses such as serum complement and oxidative stress; ii. Fur, the major transcriptional regulator of iron homeostasis; iii. FNR, the master regulator involved in the adaptation to oxygen-limited conditions; iv. the alternative sigma factor E (σE) that in pathogenic *Neisseria* species is activated in response to oxidative stress and negatively controls transcription of *aniA*, encoding the nitrite reductase (Ampattu et al., 2017).

The analysis of the genes involved in glutamate metabolism may provide a concrete example of how differences in gene regulation between meningococcal strains are directly associated with their pathogenic potential. Northern blot analysis showed differential expression of *ghdA* in different *N. meningitidis* strains, and the highest levels of *gdhA* mRNA were observed in strains belonging to the hypervirulent clonal complexes ST-32 (ET-5, serogroup B) and ST-4 (subgroup IV-1, serogroup A) (Pagliarulo et al., 2004). The underlying mechanisms were investigated, leading to the discovery that in strains expressing high levels of *gdhA* mRNA, two promoters, *gdhA* P1 and *gdhA* P2, initiate transcription of *gdhA*. In contrast, in strains expressing low levels of mRNA, *gdhA* P2 was not active due to the weak expression of *gdhR*, an associated regulatory gene, as a result of the insertion of the miniature neisserial element (nemis also known as Correia element) downstream of *gdhR* that destabilizes its mRNA. The study also showed that: i. transcription from *gdhA* P2 was maximal in complex medium during late logarithmic growth phase and in chemically defined medium when glucose instead of lactate was used as a carbon source in the presence of glutamate; ii. 2-oxoglutarate, a product of the catabolic reaction of the NADP-GDH and an intermediate of the TCA cycle, inhibits the binding of GdhR to *gdhA* P2 (Pagliarulo et al., 2004).

*N. meningitidis* is known to possess a relatively low number of transcriptional regulators compared to other bacterial species. Thirty-five are annotated in the MC58 strain genome, and of these 35, only a few have been well characterized: i. CrgA, a LysR-type regulator that is upregulated upon contact with human epithelial cells and represses its own transcription and that of the type IV pili subunits *pilE* and *pilC1*; ii. AsnC, a global transcriptional regulator that controls the response to poor nutritional conditions, which are sensed by binding of this regulator to leucine and methionine; iii. Fur, the above-mentioned global regulator of iron homeostasis, which exerts its function also indirectly controlling the expression of small regulatory RNAs; iv. the fumarate and nitrite reductase regulator FNR, which in case of oxygen limitation allows the meningococci to survive by changing the metabolism toward sugar fermentation and denitrification; v. NsrR, a repressor of a compact regulon of genes in the absence of nitric oxide; vi. HexR, an RpiR-like transcriptional regulator, HexR, that is responsible for part of the glucose-responsive regulation and affects the fitness of the meningococcus *in vivo* (Claus et al., 2007; Schielke et al., 2010; Antunes et al., 2015).

In addition to transcriptional regulators, *N. meningitidis* utilizes small regulatory RNAs that are important for meningococcal adaptation to specific environmental changes during colonization and invasive infection. Three of these transcoding noncoding RNAs have been characterized: NrrF, AniS, and NmsR. NrrF is essential for iron homeostasis, is expressed under iron-limiting conditions under the control of Fur and is known to inhibit the translation of *sdhA* and *sdhC* mRNAs, which encode subunits of succinate dehydrogenase complex, an iron-containing enzyme. AniS is part of the FNR regulon and may be responsible for the downregulation of FNR-repressed genes. NmsR downregulates mRNAs targeting *prpB* and *prpC*, encoding proteins involved in the methylcitrate cycle (Eichner et al., 2022).

Despite this relatively small number of transcriptional regulators and small regulatory RNAs, meningococcus has a broad capacity to regulate gene expression at the population level, thanks to the presence of sophisticated ON/OFF phase variation mechanisms. Specifically, the *N. meningitidis* genome is characterized by an abundance of homopolymeric DNA repeats (simple sequence DNA repeats, SSRs) that undergo stochastic and reversible mutations (insertion/deletion) at a high frequency by slipped-strand mispairing during DNA synthesis associated with DNA replication or recombination (Saunders et al., 2000; Snyder et al., 2001). The length of the SSRs, when located within a coding region, can change translation by introducing a frameshift in the reading frame, or, when located in the proximity of a promoter, can modulate transcription either by altering the promoter sequence or by switching between alternative promoter sites. This sophisticated mechanism of genetic variation of “contingency” genes is responsible for a broad phenotypic diversity at the population level and is thought to facilitate the adaptation of meningococci to dynamic environmental changes (Alexander et al., 2004; Davidsen and Tønjum, 2006; Moxon et al., 2006; Klughammer et al., 2017; Green et al., 2020). It is noteworthy that the rate of phase variation is modulated in the meningococcal population due to the presence of mutator phenotypes (see below). A population-scale comparative genomic analysis identified 277 genes and classified them into 52 strong, 60 moderate, and 165 weak candidates for phase variation. Deep-coverage DNA sequencing of single colonies grown overnight under non-selective conditions confirmed the presence of high-frequency, stochastic variation in 115 of them (Siena et al., 2016). Functional characterization of these phase-variable 115 genes demonstrated an enrichment for those encoding already known surface determinants (capsule, LOS, adhesins, capsule, nutrient-scavenging proteins) or DNA metabolism. However, among phase-variable genes, genes involved in a broad spectrum of other metabolic functions were found. In addition, most of the variable SSRs were predicted to induce phenotypic changes by modulating gene expression at a transcriptional level or by producing different protein isoforms rather than mediating on/off translational switching through frameshifts as previously known (Siena et al., 2016).

Among the phase-variable genes, there are genes encoding DNA methyltransferases (Mod) belonging to the type III restriction-modification systems. This is of particular interest because Mod proteins mediate epigenetic control (Srikhanta et al., 2005) Random, reversible mutation of simple sequence DNA repeats within the open reading frame of *mod* genes leads to frameshift mutations and ON/OFF Mod expression. This results in a different methylation pattern of the genome and modified expression of specific sets of genes under the control of specific Mod proteins. These regulons are termed “pasevarions” (phase variable regulons) and are implicated in strain differences in virulence traits (Srikhanta et al., 2005; Tan et al., 2016).

In conclusion, gene regulation is increasingly recognized as a key determinant of meningococcal virulence. In fact, virulence determinants are uniformly distributed in the *N. meningitidis* population, with no obvious differences between clonal complexes more or less associated with IMD. Therefore, their differential regulation makes the difference. Differential gene expression between hypervirulent and carrier strains affects numerous genes, many of which are involved in meningococcal metabolism, highlighting its importance in meningococcal disease, while other genes encode virulence determinants or proteins involved in genome stability and gene regulation (**Table 2**). However, there is no uniform pattern that can dichotomously distinguish hypervirulent strains from carrier strains, which indicates that meningococcal pathogenicity is complex and multifactorial.

## 8 Genome plasticity, microevolution, and hypervirulent phenotype

Several studies on *N. meningitidis*, a microorganism characterized by high genomic plasticity, have highlighted the relationship between microevolution, antimicrobial resistance, vaccine escape, and hyperinvasive phenotype (Kugelberg et al., 2008; Brehony et al., 2016; Mikucki and Kahler, 2023). The high plasticity of the genome arises from several factors, among which the most relevant appear to be: i. the natural competence of *N. meningitidis* for natural transformation; ii. The presence of sophisticated mechanisms of phase variation of “contingency” genes; iii. The presence of specific mechanisms of antigenic (and functional) variation of key determinants involved in the interaction with the host; iv. The high frequency in the meningococcal population of allelic variants coding for proteins with reduced functionality involved in DNA mismatch repair encoding proteins (MutS, MutL) and homologous recombination (RecB, RecC, UvrD) (Feil et al., 1999; Holmes et al., 1999; Richardson and Stojiljkovic, 2001; Richardson et al., 2002; Colicchio et al., 2006a).

The high propensity for genetic transformation of meningococcus, a bacterium with minimal genetic barrier to recombination and a unique, non-regulated competence system that allows it to capture DNA throughout the entire growth cycle, is such that this species presents non-clonal, but panmictic characteristics (Maiden, 1993, Maiden, 2006; Spratt and Maiden, 1999; Bentley et al., 2007). Furthermore, the mutator *mutS* and *mutL* alleles contribute to reducing the genetic barrier to recombination (Alexander et al., 2004; Davidsen and Tønjum, 2006), as it is known that MutS and MutL mismatch binding activity edit (prevent) homologous and homoeologous recombination, limiting the stability of heteroduplex intermediates, with the contribution of the RecBCD nuclease (Štambuk and Radman, 1998).

The high incidence of *mutS* and *mutL* mutator alleles in the *N. meningitidis* population is thought to be a primary factor in the adaptive evolution of meningococcus, increasing overall spontaneous mutation rates and the rate of phase variation of “contingency” genes (Saunders et al., 2000; Siena et al., 2016; Klughammer et al., 2017; Green et al., 2020). Evidence is provided that asymptomatic meningococcal carriage on the nasopharyngeal mucosal surface is facilitated by localized hypermutation in “contingency” genes, which, together with horizontal gene transfer, affects the expression of a plethora of surface determinants involved in meningococcal adhesion, metabolism, intracellular survival, intra-species and interspecies competition, and escape of immune response (Green et al., 2020).

It has been proposed that meningococcal virulence results from the accidental emergence of invasive variants during carriage (Klughammer et al., 2017). It is worth mentioning, in this regard, a study that, analyzing a laboratory accident with a *mutS*-defective mutator strain of *Neisseria meningitidis*, which was responsible for a case of IMD that fortunately evolved with antibiotic treatment until complete recovery, describes the genotypic and phenotypic modifications of the bacterium after accidental passage into humans (Omer et al., 2011). The *in vivo* passage was responsible for the modification in key phase-variable genes. In particular, compared to the parental strain, the meningococcal isolate from the patient’s blood utilized a different hemoglobin-bound iron receptor (HpuA/B) than the parental strains (HmbR), expressed different pilin variants with different adhesion properties, and showed a different LOS immunotype. The study of this episode demonstrates the dangerousness of mutant strains of meningococcus.

Based on phase variation frequencies in *hpuAB* and *hmbR*, encoding two distinct hemoglobin receptors, Richardson and Stojiljkovic (2001) identified *mutL* alleles encoding MutL variants with multiple amino acid substitutions in MutL, associated with three distinct switching phenotypes, slow, medium, and fast, together with a *mutS* allelic variant associated with fast switch and high spontaneous mutation frequency to rifampicin-resistance. In a reference strain belonging to the hypervirulent ET-37 electrophoretic type (clonal complex ST-11), 93/4286, *mutS* was found to be inactivated by IS*1106*, while *mutL* allelic variants associated with high spontaneous mutation frequency were detected among isolates belonging to ET-24 electrophoretic type (lineage 3, clonal complex ST-41/44) (Colicchio et al., 2006a), supporting the idea that the activity of the DNA mismatch repair system, in particular MutL activity, is subject to a sophisticated mechanism of modulation within the species *N. meningitidis*. Furthermore, the mutator phenotype associated with defective MutL activity was suppressed when a non-functional *recB* allele, derived from ET-37 meningococcal strains, replaced the functional *recB* allele, suggesting that in MutL-deficient strains, hypermutation mostly arises during post-replicative DNA synthesis associated with the activity of the RecBCD recombination pathway (Colicchio et al., 2006a). The high number of allelic variants in genes involved in DNA mismatch repair and RecBCD recombination, which could correspond to functional variations in the encoded proteins, could, at least in part, explain why the *N. meningitidis* population is much more diverse than that of *N. gonorrhoeae* (Vigué and Eyre-Walker, 2019), as well as the rapid evolution of hypervirulent clones.

*N. meningitidis* is a paradigmatic microorganism that exploits the high plasticity of its genome to continuously adapt to its human host by genome microevolution. The high genome plasticity arises from well-defined and interconnected factors: i. natural competence for transformation; ii. specific mechanisms of phase variation of “contingency” genes; iii. specific mechanisms of antigenic (and functional) variation; iv. high frequency of allelic variants coding for proteins with reduced functionality involved in DNA mismatch repair and RecBCD recombination. The association between microevolutionary capacity and propensity to cause IMD in several hypervirulent lineages is particularly intriguing, as it has been proposed that meningococcal virulence results from the accidental emergence of invasive variants during carriage.

## 9 Evolution of Neisseria pathogenicity: *Neisseria brasiliensis*, a new Neisseria species with key virulence determinants of *N. meningitidis*

Within a genus such as *Neisseria*, characterized by notable genomic plasticity and consequent genetic heterogeneity, a key point of concern is the emergence of species potentially pathogenic for humans and other animals. In this regard, the evolutionary history of the gonococcus, discovered by Albert Neisser in 1879, closely related to the meningococcus, can exemplify this concept.

**Figure 4:**
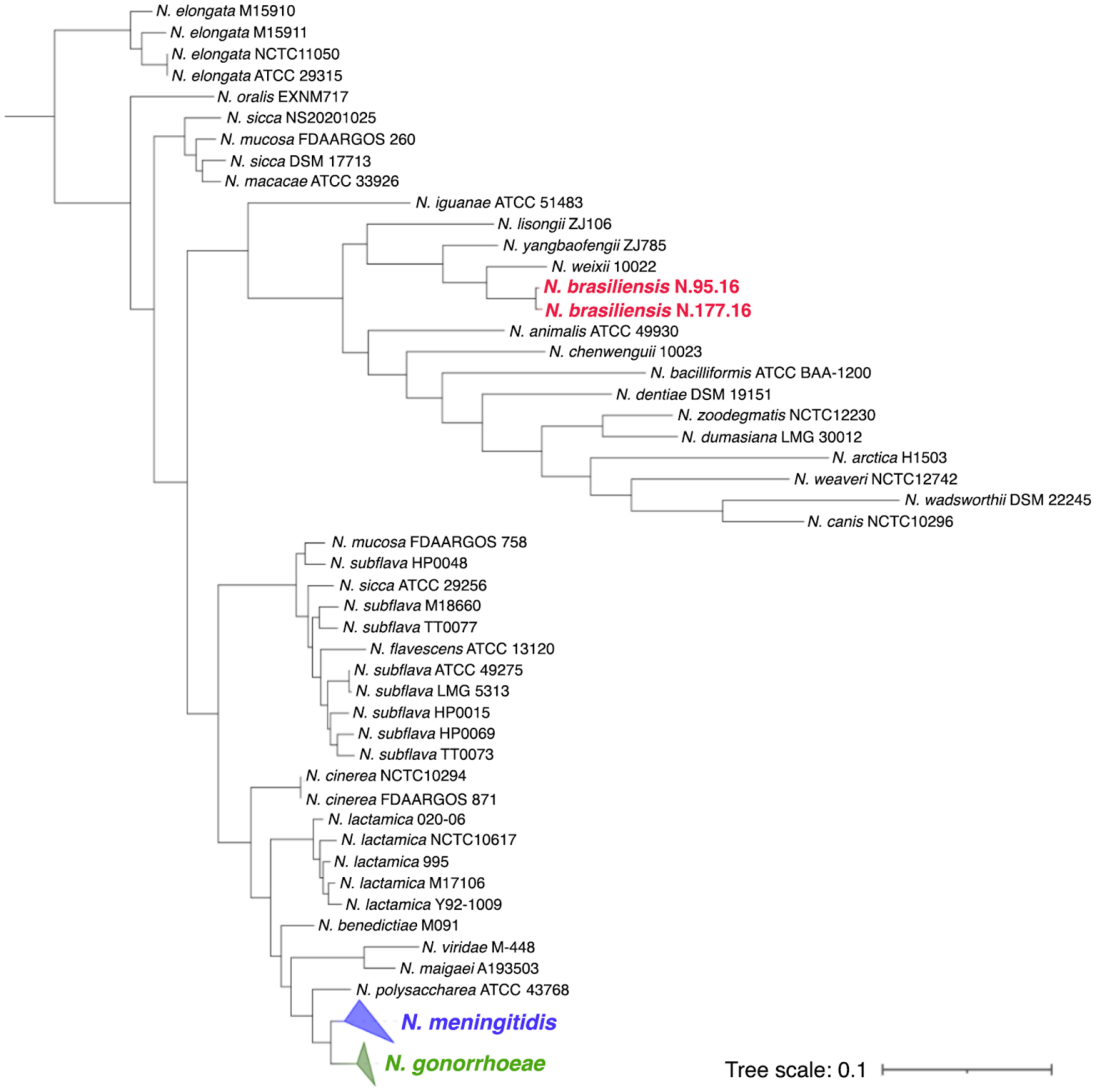
Phylogenetic tree of *Neisseria* spp. The tree is based on 16 housekeeping genes transmitted with high verticality in prokaryotes (*fusA, gatA, gatB, hisS, infB, rplA, rpsH, rpsI, rpsK, rpsM, rplB, rplF, rplN, rplW, miaB*, and rRNA 16S gene) (Gogarten et al., 2002; Nagies et al., 2020) and includes different *Neisseria* spp. The tree highlights the high phylogenetic distance between *N. brasiliensis* and *N. meningitidis.* The nucleotide sequences of the reference genes (coding region) from 276 *Neisseria* strains were obtained from the NCBI database and multiple alignment of concatenated sequences was performed using MEGA12 software via the integrated MUSCLE (MUltiple Sequence Comparison by Log-Expectation) algorithm (Kumar et al., 2024). Phylogenetic reconstruction was performed using the Maximum-Likelihood Estimation (MLE) method using the Jukes–Cantor model (Jukes and Cantor, 1969), and the results were visualized using the graphics software iTOL (itol.embl.de). The 131 N*. meningitidis* and 98 N*. gonorrhoeae* strains are not indicated. Scale bar refers to a phylogenetic distance of 0.1 nucleotide substitutions per site.

Although description of gonorrhea-like symptoms dates back to ancient civilizations like the Romans, Jews, and Arabs, genomic analysis suggests that the modern gonococcal population originated much more recently, possibly in the 16^th^ century, and was subsequently spread globally (Golparian et al., 2020). The genomic analysis provides evidence that the modern gonococcus is not as old as previously thought and shows that strains from the postmodern era (1980-twenty-first century) have evolved from strains belonging to the pre-antibiotic era (pre-1950s) and mainly form a separate clade in the tree. Furthermore, the gonococcal genome has become increasingly conserved over time, with isolates becoming increasingly clonal and the level of recombination not substantially different in different isolates (Golparian et al., 2020). This is consistent with the finding that *N. meningitidis* has acquired more of its diversity by recombination than *N. gonorrhoeae* (Vigué and Eyre-Walker, 2019), consistent with the phylogenetic reconstruction of *Neisseria* species performed with housekeeping genes transmitted with high verticality in prokaryotes (Gogarten et al., 2002; Nagies et al., 2020), showing a considerably higher radiation of *N. meningitidis* sequences compared to *N. gonorrhoeae* sequences available in the NCBI database (**Figure 4**).

Despite the high genetic similarity between *N. meningitidis* and *N. gonorrhoeae*, these two bacteria occupy distinct niches in the human host, i.e, the nasopharynx in the case of *N. meningitidis* and the urogenital tract in the case of *N. gonorrhoeae*, are responsible for distinct diseases, and are characterized by a very different infectious cycle. This is the result of a continuous adaptation of the gonococcus to its diverse ecological niche, an adaptation influenced by the use/misuse of antibiotics in more recent times, and which has mainly involved: i. modifications of some metabolic pathways (Potter and Criss, 2024); ii. modifications affecting global and pathway-specific regulatory systems (Schielke et al., 2010); iii. modifications of some determinants of the bacterial surface, involved in the interaction with the host (Schielke et al., 2010; Song et al., 2020). For example, the sulfate assimilation pathway for cysteine ​​biosynthesis and the methylcitrate pathways are in decay in the gonococcus (Potter and Criss, 2024; Talà et al., 2025), and in the 1980s, a gonococcus clade with an arginine, hypoxanthine, and uracil auxotrophy was associated with disseminated gonococcal infection (Noble et al., 1984). Furthermore, some gonococcal strains have been shown to harbor the truncated lactoferrin receptor LbpAB, despite the fitness advantage conferred by possessing a functional LbpAB in experimental infection of the human male urethra (Anderson et al., 2003). It is also interesting to note that *N. meningitidis* and *N. gonorrhoeae* share a large number of transcriptional regulators, but some, such as FadR and GdhR, have differential functions in the two bacteria (Pagliarulo et al., 2004; Schielke et al., 2010; Rouquette-Loughlin et al., 2017; Ayala et al., 2022; Mortimer, 2022). Regarding gonococcal surface structures, including type IV pili and the CEACAM-binding protein Opa, these have evolved primarily to establish and maintain colonization while inhibiting gonococcal penetration, reducing their likelihood of encountering subepithelial immune cells, explaining why most female infections are localized and asymptomatic (Song et al., 2020). This is possible due to the extraordinary ability of the gonococcus to modulate the interaction with different types of cervical epithelial cells in the female tract, using distinct mechanisms, including the modulation and control of distinct epithelial cell-cell adhesion complexes through the manipulation of host cell signaling and the ability to survive intracellularly within a gonococcal-containing vacuole (Edwards and Butler, 2011; Song et al., 2020).

A notable difference between *N. meningitidis* and *N. gonorrhoeae* is that *N. gonorrhoeae* lacks a capsule because it lacks the corresponding genes for capsule biosynthesis (Virji, 2009). This difference between the two closely related bacteria is crucial because the capsule is essential for *N. meningitidis* to cause invasive disease. Another important difference is the lack in the gonococcus of the HrpA/HrpB TPS (Talà et al., 2008; Schielke et al., 2010), which, as mentioned above, was implicated in diverse functions including adherence to epithelial cells, intracellular survival, vacuolar escape, interaction with dynein, and modulation of apoptosis/pyroptosis (Schmitt et al., 2007; Talà et al., 2008, Talà et al., 2022; van Ulsen et al., 2008; Neil and Apicella, 2009; Arenas et al., 2013), and was required for the establishment of IMD in a mouse model of meningitis (Pagliuca et al., 2024). The lack of HrpA/HrpB TPS in the gonococcus would explain why the gonococcus remains predominantly confined within a vacuole during cellular infection, unlike the meningococcus, which is able to escape, and why the meningococcus, after reaching the cytosol, induces pyroptotic pathways during cellular infection, a phenomenon not described for the gonococcus. The presence of the capsule and the HrpA/HrpB TPS represents distinctive characteristics of the meningococcus compared to the gonococcus and partly explains the different pathogenic behaviors of the two bacteria.

Two cases of human infection caused by *Neisseria brasiliensis*, a new *Neisseria* species phylogenetically distant from *N. meningitidis*, have been recently described, one of which involved bacteriemia (Mustapha et al., 2020). The first patient was a 64-year-old man from Rio Grande do Sul, Brazil, who developed congestive heart failure with bilateral pulmonary infiltrates and pleural effusion on chest X-ray in June 2016. The second patient was a 74-year-old woman with leprosy from Paraná, Brazil, who developed a polymicrobial infected ulcer on her left lower extremity in February 2016. The two cases were separated in time and by a distance of more than 400 km and had no known epidemiological link (Mustapha et al., 2020). Nevertheless, *N. brasiliensis*, a species not generally associated with human disease, was able to cause systemic disease in both cases. Despite the comorbidities of the patients, it is unusual for this bacterium to cause disease, suggesting that it may have acquired some virulence factors.

Interestingly, the two isolates (N.95–16 isolates from patient 1 and No.177–16 from patient 2) tested positive by slide agglutination for *N. meningitidis* capsular groups (ABCEWXYZ), and real-time PCR identified isolate 1 as *N. meningitidis* capsular group X and isolate 2 as capsular group B. Genomic analysis of the two isolates confirmed the presence of capsular biosynthetic genes in these two isolates (Mustapha et al., 2020). In particular, isolate N.95–16 contained *csxABC* genes that shared 98% amino acid identity with the meningococcal serogroup X reference strain α388, while isolate N.177–16 contained *cssABC-csb* genes that shared 99% amino acid identity with the meningococcal serogroup B reference strain H44/76 (Mustapha et al., 2020). The presence of serogroup B and X capsular biosynthetic genes is of particular interest because these genes are normally associated with IMD, and detection of these genes in *N. brasiliensis* could provide us with clues about the transfer of the capsular locus even among *Neisseria* that are phylogenetically and ecologically unrelated to *N. meningitidis*. In addition, the available genome sequences of *N. brasiliensis* N.95–16 and No.177-16 (Mustapha et al., 2020) revealed the presence of genes (MRN37431.1; MRN37432.1; QGL24436.1) encoding proteins with considerable protein sequence identities with *N. meningitidis* HrpA and HrpB.

These findings offer us the opportunity to continuously and directly analyze the evolution of *Neisseria* pathogenicity, and on the other hand, suggest the need to activate continuous surveillance also for *Neisseria* not normally associated with human colonization, in order to better define their pathogenic and evolutionary potential.

## 10 Conclusions

Despite numerous research studies and resources invested, the question of what determines the transition from pharyngeal colonization to IMD, which has been raised since Weichselbaum’s meningococcus, the main agent of epidemic cerebrospinal meningitis, was identified (Weichselbaum, 1887), remains without a definitive answer. Answering this question has become essential to further improve prevention and treatment strategies for meningitis and meningococcal sepsis. In fact, despite the availability of modern vaccines and effective therapeutic protocols, IMD continues to claim victims worldwide. WHO estimates that 500,000 cases of IMD occur each year, approximately 50,000 of which are fatal (Findlow et al., 2025; Poulikakos et al., 2025; Shah et al., 2025). Understanding the intracellular phase of the *N. meningitidis* infectious cycle could provide useful information to address the molecular mechanisms underlying the transition from asymptomatic (or oligosymptomatic) nasopharyngeal colonization to IMD. What is increasingly emerging from the literature is that, although it is traditionally considered an extracellular pathogen, the meningococcus engages in complex interactions with host cells in the intracellular microenvironment. These interactions involve signal transduction, membrane trafficking, cytoskeleton, metabolic cross-talk, and control of programmed host cell death. The intracellular phase of meningococcal infection may, in fact, be relevant for IMD pathogenesis. Emerging evidence indicates that this phase can be a key step for both NEB and BBB crossing, enabling bacterial dissemination into the host. The intracellular environment can be used to hide from the complement system and from innate immune mechanisms in the early phase of the infection. Moreover, intracellular meningococci induce a cellular response, such as caspase-3 activation, that participates both in the inflammatory state and in the disruption of cell-to-cell junctions. This indicates that a further role of the intracellular phase may be to favor the paracellular crossing of host barriers by the bacteria. Another important point is the considerable genetic variability within the meningococcal population, which is reflected in marked differences between the various strains, even those belonging to hypervirulent lineages, in their mechanisms of interaction with the host cell and microevolution. For instance, different meningococcal strains differ in their capability to evoke inflammation and exploit different routes to cross the NEB and BBB. These differences could explain, at least in part, some inconsistencies found among various studies, attributable to the different strains used. Caution is therefore advised when drawing general conclusions from studies conducted on a single strain or a single hypervirulent lineage.

Although there is now a fairly extensive literature confirming that the intracellular phase is crucial in meningococcal pathogenesis, there are important limitations that this article seeks to highlight. First, as mentioned above, numerous studies have been conducted with phylogenetically distant meningococcal strains, which exhibit different behaviors even in comparable experimental systems. This is due to the considerable genetic and phenotypic variability of the meningococcus. Furthermore, the results of some studies, even when using similar strains, are difficult to compare due to the very different experimental settings. A more systematic use of control reference strains and a better standardization of experimental settings could help us obtain more consistent results and also to better highlight the differences existing between the various strains and their specificities.

Second, it should be noted that much of what we know about the intracellular lifestyle of *N. meningitidis* comes from studies conducted on single or co-cultured cell lines that only remotely mimic real *in vivo* conditions. On the other hand, the use of animal models to study invasive meningococcal disease, in addition to presenting the obvious difficulties in investigating aspects of the extracellular/intracellular phase of meningococcal infection, is hampered by the fact that meningococcus has a very narrow host specificity, essentially restricted to humans. Furthermore, the use of animal models is limited for ethical reasons by increasingly stringent regulations. The use of alternative models to study meningococcal pathogenicity outside of a living organism, including organoids, tissue cultures, and *ex vivo* organ culture systems, could help us confirm observations made in cell line infection studies using traditional systems.

Third, the experimental settings used to study the intracellular/extracellular phase of meningococcal infection ignore crucial factors such as host genetics, age, environment, and lifestyle, and only very limited information is available on the interaction between *N. meningitidis* and other microorganisms and viruses in the nasopharynx. This information is limited to some observational studies and a few experimental studies involving co-culture or co-infection experiments. Further information on these aspects is essential to understand the dynamics of meningococcal infection and the main factors leading from asymptomatic colonization to disease onset, as well as the evolution of the pathogenic phenotype in *Neisseria*, in line with the idea that microbial pathogenicity is a multifactorial and complex trait, as indicated by René Dubos in the mid-1990s, who also challenged the then-dominant notion of bacterial fixity and urged bacteriologists to take note of the plasticity of bacteria in order to be ready to deal with them (Dubos, 1955, Dubos, 1959).
